# Polygoni Multiflori Radix Praeparata polysaccharides enhance gut health and mitigate ischemic stroke by regulating SCFA and amino acid metabolism in gut microbiota

**DOI:** 10.3389/fphar.2025.1580055

**Published:** 2025-05-22

**Authors:** Lingyu Ruan, Zhennan Wang, Mengyun Zheng, Qi Zheng, Qing Qing, Hongyan Lin, Yuheng Tao, Liqun Wang, Junsong Wang, Wenhao Ge

**Affiliations:** ^1^ School of Pharmacy & School of Biological and Food Engineering, Changzhou University, Changzhou, China; ^2^ School of Pharmacy, Modern Industrial College of Traditional Chinese Medicine and Health, Lishui University, Lishui, China; ^3^ Center of Molecular Metabolism, Nanjing University of Science and Technology, Nanjing, China; ^4^ The Second People’s Hospital of Changzhou, The Third Affiliated Hospital of Nanjing Medical University, Changzhou, China; ^5^ Changzhou Medical Center, Nanjing Medical University, Changzhou, China

**Keywords:** polygoni multiflori radix praeparata, polysaccharides, acute ischemic stroke, gut microbiota, metabolomics

## Abstract

**Background and purpose:**

Ischemic stroke (IS) is the most common type of stroke, known for its high rates of morbidity, disability, mortality, and recurrence. Polygoni Multiflori Radix Praeparata (PM), a traditional Chinese medicinal tonic, is frequently used for treating IS. Its polysaccharides (PMP) are acknowledged for their hepatoprotective, immunomodulatory, and neuroprotective properties. However, the effectiveness and mechanisms of PMP remain inadequately understood. This study seeks to evaluate the impact of PMP on IS and clarify the potential mechanisms involved.

**Methods:**

PMP was obtained by water extraction, alcohol precipitation, Sevage deproteinization, and dialysis. Its molecular weight, monosaccharide composition, and FT-IR spectrum were characterized. IS was established by middle cerebral artery occlusion/reperfusion (MCAO/R) in male SD rats. Neurological assessment, histopathology, and protein factors detection assessed PMP’s effectiveness. Further, ^1^H NMR based metabolomics and 16S rRNA gene sequencing examined gut microbiota metabolites and profiles, respectively, in order to elucidate the underlying mechanisms from the perspective of the gut-brain axis.

**Results:**

PMP significantly improved the neurologic functions and reduced the cerebral infarction volume in MCAO/R rats. Cerebral ischemia/reperfusion (I/R) injured brain and also affected intestine, leading to gut barrier disruption. PMP could lower the levels of inflammatory cytokines, such as TNF-α and IL-1β, and boost tight junction protein both in brain and intestinal tissues. Metabolomic analysis shows PMP raised intestinal levels of SCFAs (butyrate, propionate) and beneficial amino acids, thus improving disrupted carbohydrate and amino acid metabolism. 16S rRNA gene sequencing revealed that the abundance of *Bifidobacterium*, *Muribaculaceae*, and *Lactobacillus* was reversed after PMP intervention. The family *Bacteroidaceae* and the genus *Bacteroides* correlated positively with amino acids, and might contributed to the SCFAs production. While the family *Peptostreptococcaceae* and the *genus Romboutsia* correlated negatively with SCFAs, might related to the poor prognosis of IS.

**Conclusion:**

PMP markedly enhanced SCFAs and amino acid metabolism, as well as the proliferation of beneficial gut microbiota, thereby promoting intestinal health and barrier integrity, and thus relieving MCAO/R induced I/R injury both in brain and intestine. Our study substantiates the potential of PMP as a prebiotic health supplement in clinical settings for the prevention and treatment of IS.

## 1 Introduction

Ischemic stroke (IS) represents the predominant form of cerebrovascular disease, accounting for approximately 80%–85% of all stroke incidents. This condition arises from cerebral artery thrombosis or embolism, leading to an inadequate blood supply to specific brain regions, with a notable prevalence in the middle cerebral artery territory (Tobin et al., 2014; Yenari et al., 2014). A classic of traditional Chinese medicine (TCM), “LingShu Chapter Shizhong”, states: treating the lower part for curing diseases located on the upper part. This theory implicates the close interactions between gut and brain in physiology and pathology. Statistical evidence indicates that 50% of stroke patients are likely to encounter clinical gastrointestinal complications (Kumar et al., 2010). Furthermore, individuals with concurrent cerebrovascular disease and gastrointestinal complications generally exhibit a poorer prognosis (Huang and Xia, 2021; Xiang et al., 2020). Substantial evidence suggests that ischemic brain injury can disrupt the homeostasis of intestinal microbiota, resulting in various gastrointestinal responses, including intestinal barrier dysfunction and bacterial translocation, which may further precipitate systemic inflammatory responses (Hanscom et al., 2021).

In recent years, significant attention has been directed towards the role of intestinal microbiota. The gut microbiota is crucial in initiating neuroinflammatory responses following brain injury, which may either exacerbate stroke or confer protection (Clarke et al., 2015). The human gut harbors approximately 10^14^ microorganisms, a quantity that surpasses the total number of human cells by an order of magnitude, with bacteria constituting a major component. The colon alone hosts approximately 300–500 distinct bacterial species. Alterations in gut microbiota have been identified as novel risk factors for stroke, with patients exhibiting significant dysbiosis in both microbial diversity and composition (Yin et al., 2015). For instance, the enrichment of *Enterobacteriaceae* has been identified as an independent risk factor for poor prognosis in stroke patients, exacerbating cerebral infarction through the promotion of systemic inflammation (Xu et al., 2021). Additionally, the gut microbiota possesses the capability to metabolize various food and drug molecules, producing a spectrum of metabolites including short-chain fatty acids (SCFAs), indole derivatives, and polyamines. These metabolites are implicated in the regulation of bodily homeostasis and the progression of disease (Feng et al., 2018). Consequently, the adjustment of the deviated gut microbiota could be a valuable strategy for the prevention and treatment of IS.

Polygoni Multiflori Radix Praeparata (PM), derived from the raw root of a renowned traditional Chinese herb *Polygonum multiflorum* thunb., has been included in the fourth batch of Chinese herbal medicines approved by the Ministry of Health of the People’s Republic of China for use as dietary supplements. PM is commonly utilized in pharmaceutical formulations, such as classical formula “Peiyuan Tongnao Capsule”, “antithrombotic rebuilding pill” and “awakening brain rebuilding capsule”, for the management of conditions such as stroke and cerebral thrombosis (Bai et al., 2019a; Bai et al., 2019b). Its principal therapeutic actions encompass the augmentation of “qi” and the facilitation of blood circulation (Bai et al., 2019b). In preclinical trials, PM alleviated ischemia/reperfusion (I/R) injury by enhancing energy metabolism, reducing oxidative stress, and modulating inflammatory pathways such as Nrf2 (Li et al., 2018). It also improves cognitive function in Alzheimer’s disease models through reshaping gut microbiota composition—elevating beneficial *Lactobacillus* and reducing harmful bacteria—which in turn alleviates inflammation and neuronal senescence (Liu et al., 2025). In PM, polysaccharide is the main active ingredient. Studies focusing on Modified Dioscorea Pills demonstrated that the PM polysaccharide (PMP) is one of the major ingredients responsible for its cognitive function and neuroplasticity improvments (Li et al., 2020). Polysaccharides are intricate macromolecules consisting of at least ten monosaccharides linked by glycosidic bonds (Xie et al., 2016). Natural polysaccharides have the potential to modulate the composition and metabolism of gut microbiota, thereby restoring intestinal mucosal barrier function and enhancing immune system activity (Huo et al., 2022). While there is indeed limited direct research on PMP in the specific context of gut microbiota and its metabolism regulation in IS. Therefore, we investigated whether PM Polysaccharides exert a therapeutic effect on IS induced by middle cerebral artery occlusion (MCAO) surgery in rats, and applied ^1^H NMR based metabolomics and 16S rRNA sequencing to reveal the underlying mechanisms. The findings indicated that PMP ameliorate cerebral I/R injury by modulating the composition of intestinal microbiota, correcting metabolic abnormalities, regulating barrier proteins, and inhibit the inflammation response.

## 2 Materials and methods

### 2.1 Preparation and qualitative analysis of PM polysaccharides

Processed PM (NT.23020718) was procured from Nanjing Tongrentang, originating from Liupanshui, Guizhou. PM power (100 g) was introduced into distilled water (2 L), maintaining a solid-to-liquid ratio of 1:20. The mixture was extracted by reflux heating for 2 h. The resultant filtrate was collected, and the extraction process was repeated three times. The combined extracts were subsequently concentrated under reduced pressure, then absolute ethanol was added to a final concentration of 80%. The mixture was left to precipitate at 4°C overnight. The crude PMP precipitate was then collected and washed multiple times with ethanol and acetone. Further, the crude PMP underwent redissolution and deproteinization using the Sevage method. This was followed by dialysis against distilled water for 48 h. The resultant solution was then concentrated and subjected to lyophilization to yield PMP.

The sugar content was determined by the phenol-sulfuric acid method using D-glucose as a standard. The BCA assay was used to determine the content of total protein. Ash content was detected at 400°C for 8 h by using a muffle furnace. High Performance anion Exchange Chromatograph (Dionex ICS-5000, Thermo Fisher, United States) with a CarboPac PA10 anion Exchange column (2 mm × 250 mm) was utilized to determine the monosaccharide composition, using glucose, xylose, cellobiose and arabinose as standard samples. Fourier-transform infrared spectroscopy (FT-IR) analysis using IS50 ThermoFisher was performed to confirm the characteristic absorption peaks, with a detection range of 4,000–400 cm^−1^.

### 2.2 Establishment of MCAO model

Male Sprague-Dawley (SD) rats (170 ± 10 g) were procured from Nanjing Qinglongshan Animal Breeding Farm and maintained under a standard light-dark cycle (12 h: 12 h, temperature 25°C) with *ad libitum* access to food and water. All animal experiments received approval from the Animal Care and Use Committee of Changzhou University (Approval No. 20230.1002) and were conducted at the Laboratory Animal Center of Nanjing University of Science and Technology. The welfare of the animals and the laboratory protocols strictly complied with established guidelines for the care and use of laboratory animals.

After a 12-h fasting period, the MCAO procedure was initiated by inducing inhalation anesthesia with 5% isoflurane and maintained by 1.0%–1.5% isoflurane using an anesthesia machine (EZVET F710, RWD Life Science). Then, the common carotid artery (CCA) was ligated, and a monofilament (Pingdingshan Yushun Biotech, Henan, China) was inserted through the external carotid artery (ECA) and carefully advanced to occlude the ipsilateral middle cerebral artery. Following 1-h occlusion period, reperfusion was achieved by removing the monofilament (Azedi et al., 2019). Neurological function was assessed on days 1, 3, and 7 utilizing the Zea Longa 5-level scoring system (Longa et al., 1989). The criteria for this scoring system are as follows: a score of 0 indicates no neurological impairment; a score of 1 indicates the extension of the contralateral forepaw; a score of 2 indicates circling to the contralateral side; a score of 3 indicates falling to the contralateral side; and a score of 4 indicates a loss of consciousness and an inability to walk independently. Additionally, the whole brains of euthanized rats were taken into 2 mm tissue sections and stained with 2% 2, 3, 5-tribenzotetraazonium chloride (TTC) solution for 20 min at 37°C, showing the degree of ischemic infarction. ImageJ software was used to calculate the area of cerebral infarction (O’Donnell et al., 2004).

### 2.3 Animal experiments design

**FIGURE 1:**
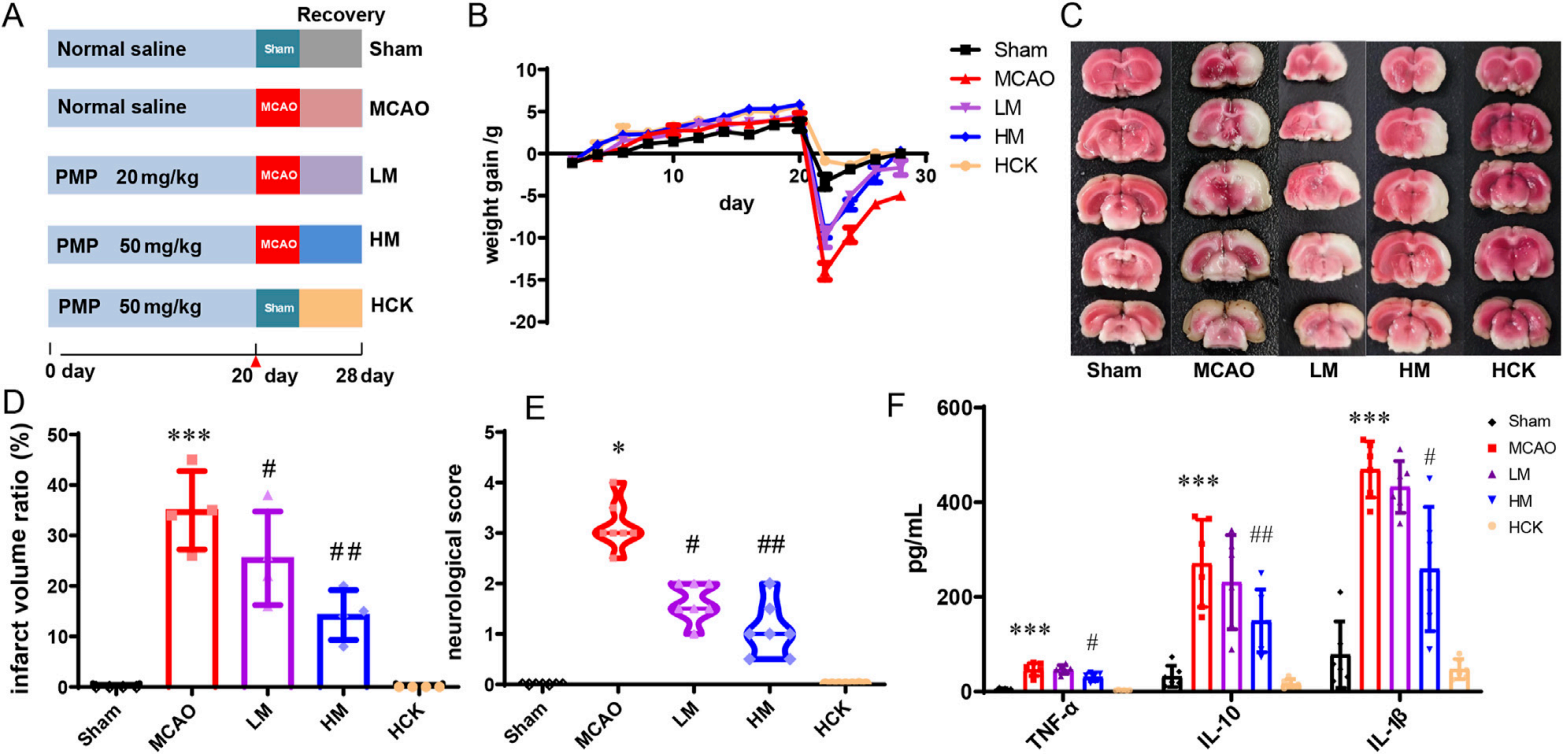
PMP pre-administration has a protective effect on brain I/R injury in MCAO rats. **(A)** Experimental grouping and flow chart. **(B)** Diagram of body weight changes of rats in each group. **(C–E)** TTC staining, cerebral infarction volume (%/hemisphere), and neurobehavioral deficit scores of brain sections, n ≥ 3. **(F)** Serum levels of inflammatory cytokines TNF-α, IL-10, and IL-1β in rats, n ≥ 3. *p < 0.05, **p < 0.01 and ***p < 0.001, MCAO group vs. Sham group; #p < 0.05, ##p < 0.01 and ###p < 0.001, dosing group vs. MCAO group.

To investigate the efficacy of PMP on IS, both high- and low-dose PMP interventions were administered. Following a 1-week acclimation period, SD rats were randomly assigned to five groups, each comprising 12 rats, including a control (Sham) group receiving normal saline, a MCAO group receiving normal saline, a low-dose group (LM) receiving 20 mg/kg PMP, a high-dose group (HM) receiving 50 mg/kg PMP (Figure 1A). And a high-dose positive control group rats (HCK) receiving 50 mg/kg PMP were included to evaluate the potential toxicity of high intake of PMP. After 21 days’ PMP/saline administration, rats of MCAO, LM and HM groups underwent MCAO with reperfusion following 1 h of ischemia, and rats of Sham and HCK group underwent sham-operation with no ischemia and reperfusion. Subsequent interventions with PMP were lasted over a period of 7 days following the modeling. Samples from the serum, brain, distal colon and colonic feces, were collected for subsequent analysis.

### 2.4 H&E staining and immunohistochemistry

Brain and colon tissues were harvested and subsequently fixed in a 4% formaldehyde solution. The tissues were then dehydrated using a graded ethanol series ranging from 70% to 100% and embedded in paraffin. The embedded samples were sectioned into 5 μm thick slices using a rotary microtome. These sections were stained with hematoxylin and eosin (H&E) following the standard protocol. The immunohistochemistry experiments were performed according to standard procedures. Firstly, brain and colon sections were soaked in 3% H_2_O_2_ for 10 min to remove endogenous catalase, followed by a water rinse. Then, the sections were incubated in 3% FBS for 1 h at room temperature, followed by PBST washing three times. After incubation, the sections were incubated in respective primary antibodies at 37°C for 1 h, followed by three washes with PBST. Subsequently, the corresponding secondary antibodies were incubated with the sections at 37°C for 30 min. Finally, all sections were subjected to diaminobenzidine and stained with hematoxylin for 1–3 min. After dried completely, the immunostained sections were observed and photographed.

### 2.5 Western blot and ELISA assay

The proteins for Western blot (WB) were extracted from the ischemic region of brain and also colon with protein extraction kits according to the instructions. The protein concentrations were determined using the BCA Protein Assay Kit (Thermo Scientific, United States, LOT: XJ357486). Protein samples were subjected to fractionation using 10% SDS-PAGE and subsequently transferred onto PVDF membranes. The membranes were then blocked in Tris-buffered saline containing 1% Tween-20 (TBST) and 5% skim milk at room temperature for 2 h, and then incubated overnight at 4°C with primary antibodies. Finally, the membranes were rinsed several times with TBST and subsequently incubated with the corresponding secondary antibodies for 1 h at room temperature. The antibody-protein complexes on PVDF membranes were visualized utilizing Enhanced Chemiluminescence (ECL) kits (Tanon^TM^High-sig, China), followed by image acquisition using the Tanon Imaging System (Tanon 5200, China). The concentrations of the cytokines IL-1β, TNF-α, and IL-10 in the serum of SD rats were quantified using enzyme-linked immunosorbent assay (ELISA) kits (Absin Bioscience Inc., Shanghai, China), following the protocols provided by the manufacturer.

### 2.6 ^1^H NMR based metabolomics analysis

Sample preparation for ^1^H NMR testing was performed as reported before (Meng et al., 2023). The fecal samples were weighed and subsequently dissolved in a tenfold volume of phosphate buffer (100 mL D_2_O containing 2.07 g Na_2_HPO_4_, 2.41 g NaH_2_PO_4_, and 10 mg sodium 3-(trimethylsilyl)propionate-2,2,3,3-d4 (TSP)). The resulting mixture was briefly vortexed, followed by the extraction of intestinal contents through six freeze-thaw cycles. The extract was then centrifuged at 16,000 g for 10 min at 4°C. A 500 μL aliquot of the supernatant was utilized for ^1^H NMR spectroscopy testing, with TSP serving as the chemical shift reference.

^1^H NMR spectroscopy was conducted at 298 K using a 500 MHz Bruker Avance spectrometer (AvanceIII, Bruker, Switzerland)) with the CPMG pulse sequence to improve small metabolite detection and reduce water peak interference. Samples were manually loaded, and 32 scans were taken over a 10,000 Hz spectral width with 32k data points. Before analyzing, the spectroscopy was baselined and phase-corrected using Topspin 2.1 (Bruker GmbH, Karlsruhe, Germany) and referenced to 0 at the highest point of the TSP outgoing peak. The processed spectral data were then imported into MestReC (version 3.7.4, Mestrelab Research SL) and converted to ASCII file format for further analysis. The ASCII files were imported into R (http://cran.r-project.org/). Water peak at 4.50–5.50 ppm was removed to prevent their influence on the spectra, which were then integrated at 0.005 ppm intervals. Representative spectra were selected using the Chenomx NMR suite 7.5 library, supplemented by the Human Metabolome Database (HMDB) and the Madison-Qingdao Metabolics Consortium Database (MMCD), to match metabolite fingerprints. The identified metabolites were seen in Supplementary Figure S1 and detailed in Supplementary Tables S1, S2. Normalization was done with the ProbNorm function to remove global concentration differences, and multivariate statistical analyses, including principal component analysis (PCA) and orthogonal signal correction-partial least squares-discriminant analysis (OSC-PLS-DA), were performed to identify the variance between the modeling, sham and treatment groups. Differential metabolite fold changes were calculated using the Benjamini & Hochberg method (Ferreira, 2007). The KEGG database (http://www.kegg.jp/) and the MetaboAnalyst website (https://www.metaboanalyst.ca/home.xhtml) were used to further clear and definite these altered metabolites in various metabolic pathways and perform Enrichment pathway analysis.

### 2.7 16S rRNA gene sequencing

The amplification and sequencing of the 16S rRNA gene were conducted by Kidio (Guangzhou, China). Initially, DNA was extracted, and its quality and concentration were assessed. The extracted DNA underwent real-time PCR to amplify the V3-V4 region of the 16S rRNA gene. The PCR product was subsequently purified using AMPure XP Beads and quantified with Qubit 3.0. Sequencing was carried out utilizing the Illumina DNA Prep Kit (Illumina, CA, United States). Library quality was evaluated using the ABI StepOnePlus Real-Time PCR System (Life Technologies, United States), and sequencing was performed in PE250 mode.

LEfSe (Linear discriminant analysis Effect Size) is a statistical tool used in 16S rRNA sequencing data analysis to identify taxa that differ significantly between groups. It combines the Kruskal-Wallis rank-sum test for class comparison with linear discriminant analysis (LDA) to estimate the effect size of these differences, revealing biomarkers with biological relevance (Segata et al., 2011). PICRUSt2 predictive analysis uses phylogenetic placement of 16S rRNA gene sequences to infer the functional capabilities of microbial communities. It predicts the abundance of gene families and pathways by mapping 16S sequences to reference genomes and applying hidden-state prediction algorithms, enabling functional profiling without full metagenomic data (Douglas et al., 2020). Spearman’s correlation analysis was conducted at the genus and family level to explore the correlation between the levels of altered gut microbiota and fecal metabolites.

### 2.8 Statistical analysis

All the experimental data from at least three samples are expressed as the mean ± standard deviation (SD). Statistical analyses were conducted using GraphPad Prism software, version 9.3.0 (463). One-way ANOVA with Newman–Keuls post-hoc test was used to evaluate the significance of all pairs. A *p*-value of less than 0.05 was considered to indicate statistical significance.

## 3 Results

### 3.1 PMP alleviates I/R brain injury in MCAO rats

The water-soluble polysaccharide of processed PM was extracted using hot water extraction method followed by an 80% ethanol precipitation, resulting in a yield of 5%. Chemical analysis results (Table 1) showed that the contents of polysaccharide, protein and Ash in PMP were 84.7%, 5.30%, and 1.8%, respectively. The analysis of the monosaccharide composition revealed that PMP is comprised of glucose, xylose, cellobiose, and arabinose. The IR spectrum of PMP (Supplementary Figure S2) exhibited strong absorption peaks at 3,405, 2,924, 1,599∼1,327, and 1,153∼1,025 cm^−1^, corresponding to the vibrations of O–H stretching vibration, C–H stretching vibration, C–H deformation vibration and C–O/C–O–C stretching vibration overlap, respectively. Additionally, the absorption band centered at 934 cm^−1^ represents the characteristic dominant structure of the pyranose form, confirming that the glycosidic bond is of the β-type. These results indicated that PMP mostly consists of pyranoses with β-glycosidic bond.

**TABLE 1:** The chemical analysis of PMP.

Detection items	Results
Extraction yield (%)	5%
Polysaccharide content (%)	84.70%
Protein content (%)	5.30%
Ash content (%)	1.8%
Monosaccharide composition	Glucose (72.8%), xylose (4.7%), cellobiose (1.8%), arabinose (2.7%)

**FIGURE 2:**
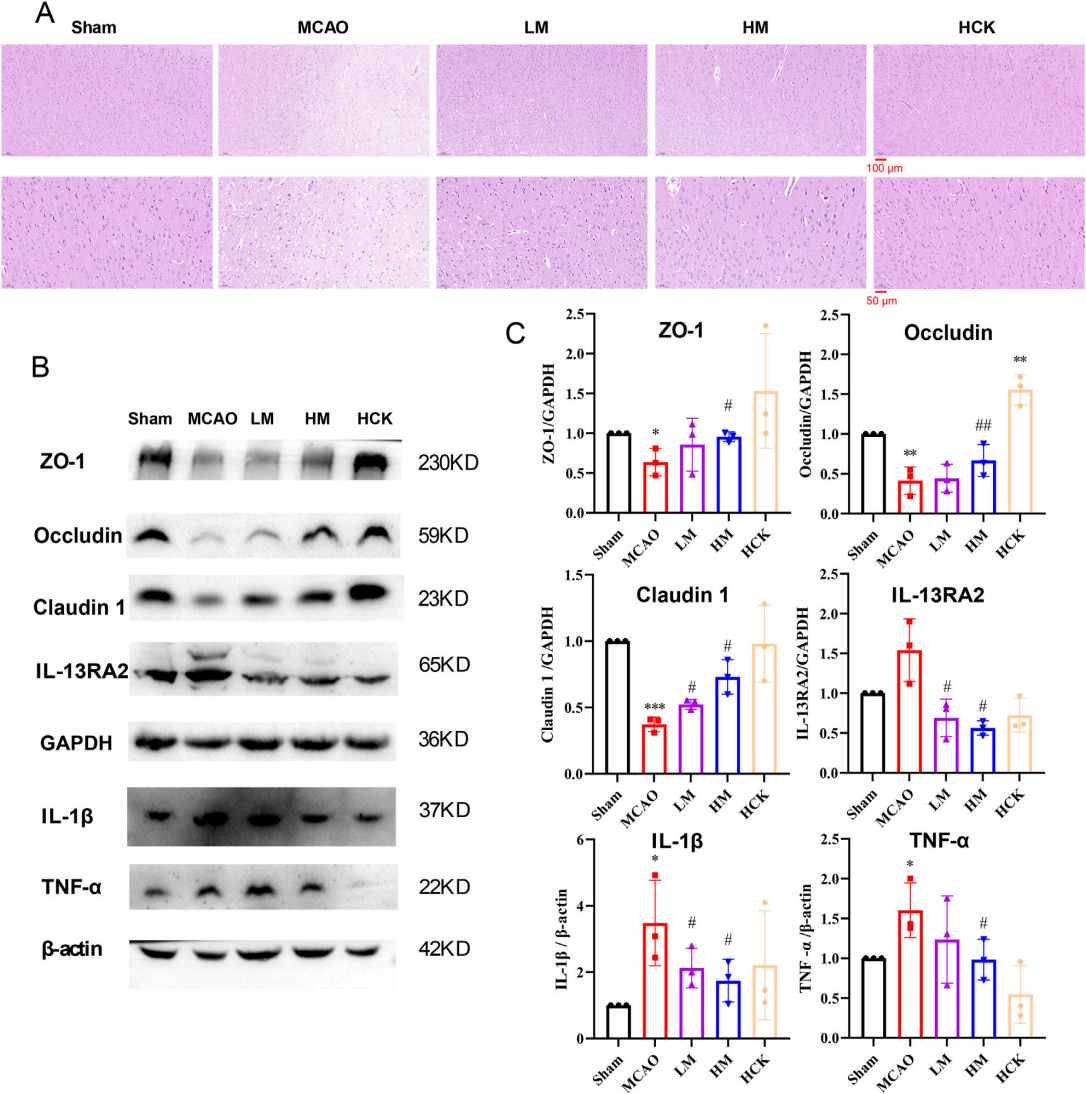
PMP reduced brain tissue damage, preserved barrier proteins, and suppressed inflammatory factor expression. **(A)** H&E staining of representative brain sections. n = 3. **(B, C)** WB result chart and gray value statistical chart of ischemic hemiencephale. n = 3. *p < 0.05, **p < 0.01 and ***p < 0.001, MCAO group vs. Sham group; #p < 0.05, ##p < 0.01 and ###p < 0.001, dosing group vs. MCAO group.

As illustrated in Figure 1B, following a 21-day pre-administration and a 7-day treatment period with PMP, the rats in the HM group exhibited a more favorable prognosis, evidenced by faster weight gain following MCAO surgery compared to the MCAO group. Furthermore, as depicted in Figures 1C–E, PMP intervention significantly reduced total infarct volume and neurobehavioral deficit scores. Additionally, PMP significantly decreased serum levels of TNF-α, IL-10, and IL-1β in a dose-dependent manner (Figure 1F). Histological examination of HE-stained brain sections revealed that MCAO resulted in darkly stained and condensed neuronal nuclei, shrunken cell bodies, and vacuolated surroundings, whereas PMP treatment improved these results (Figure 2A). In Figures 2B,C, MCAO rats showed reduced expression of cerebral tight junction proteins (occludin, claudin, and ZO-1), key components of the blood-brain barrier (BBB). Claudin levels rose significantly in the LM and HM groups, l, but occludin and ZO-1 stayed low. Pro-inflammatory cytokines TNF-α and IL-1β were significantly upregulated, but high-dose PMP significantly inhibited their production. Interestingly, IL-13RA2, a type of IL-13 receptor, was upregulated in MCAO rats. In the HCK group, PMP did not affect structural protein levels but partially inhibited inflammatory factor expression (Figure 2B).

### 3.2 PMP alleviates I/R-induced intestinal injury in MCAO rats

**FIGURE 3:**
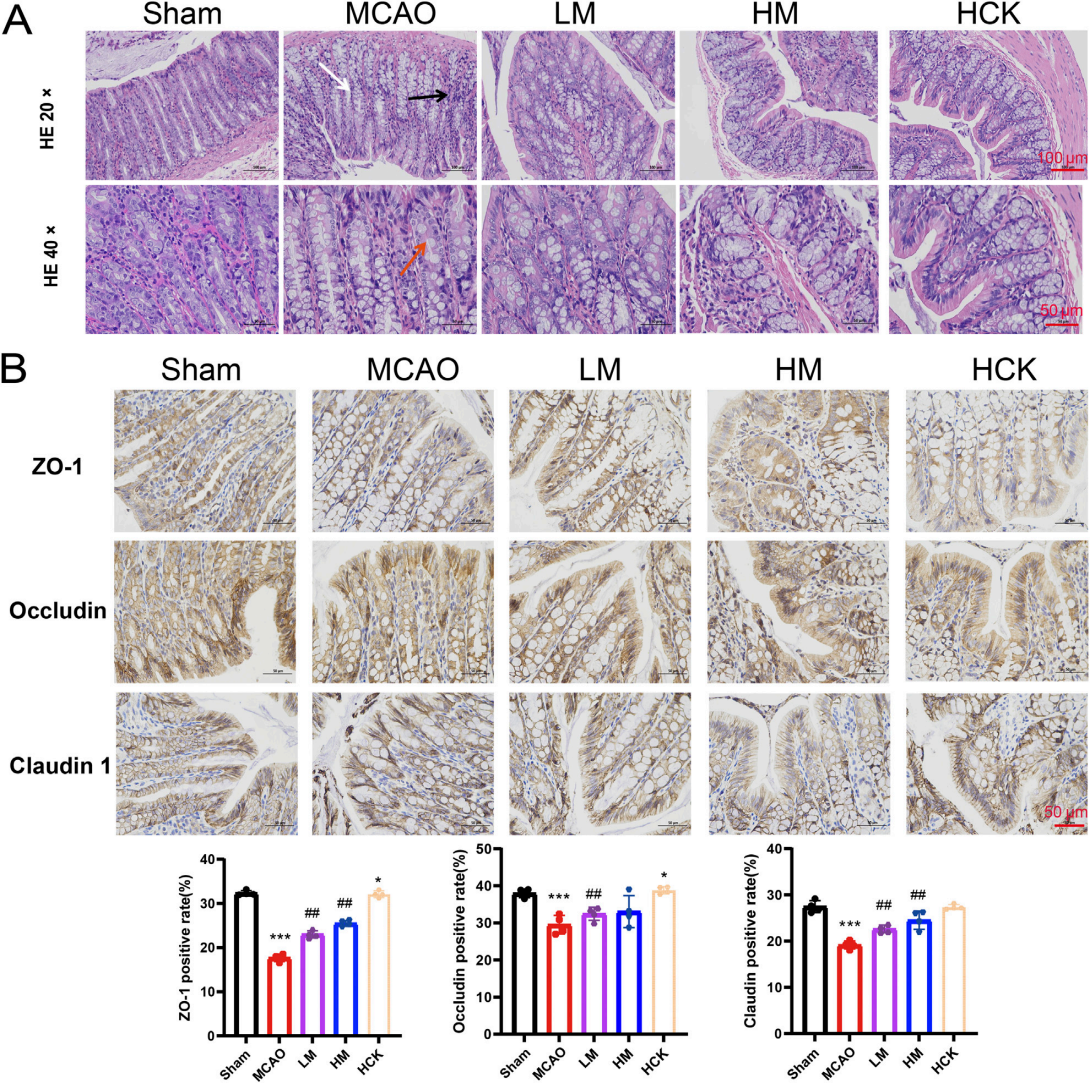
PMP has an ameliorating effect on colon injury in MCAO rats. **(A)** Representative micrographs of H&E staining in the colon. **(B)** Representative immunohistochemical images and statistical maps of tight junction protein. n = 4. *p < 0.05, **p < 0.01 and ***p < 0.001, MCAO group vs. Sham group; #p < 0.05, ##p < 0.01 and ###p < 0.001, dosing group vs. MCAO group.

**FIGURE 4:**
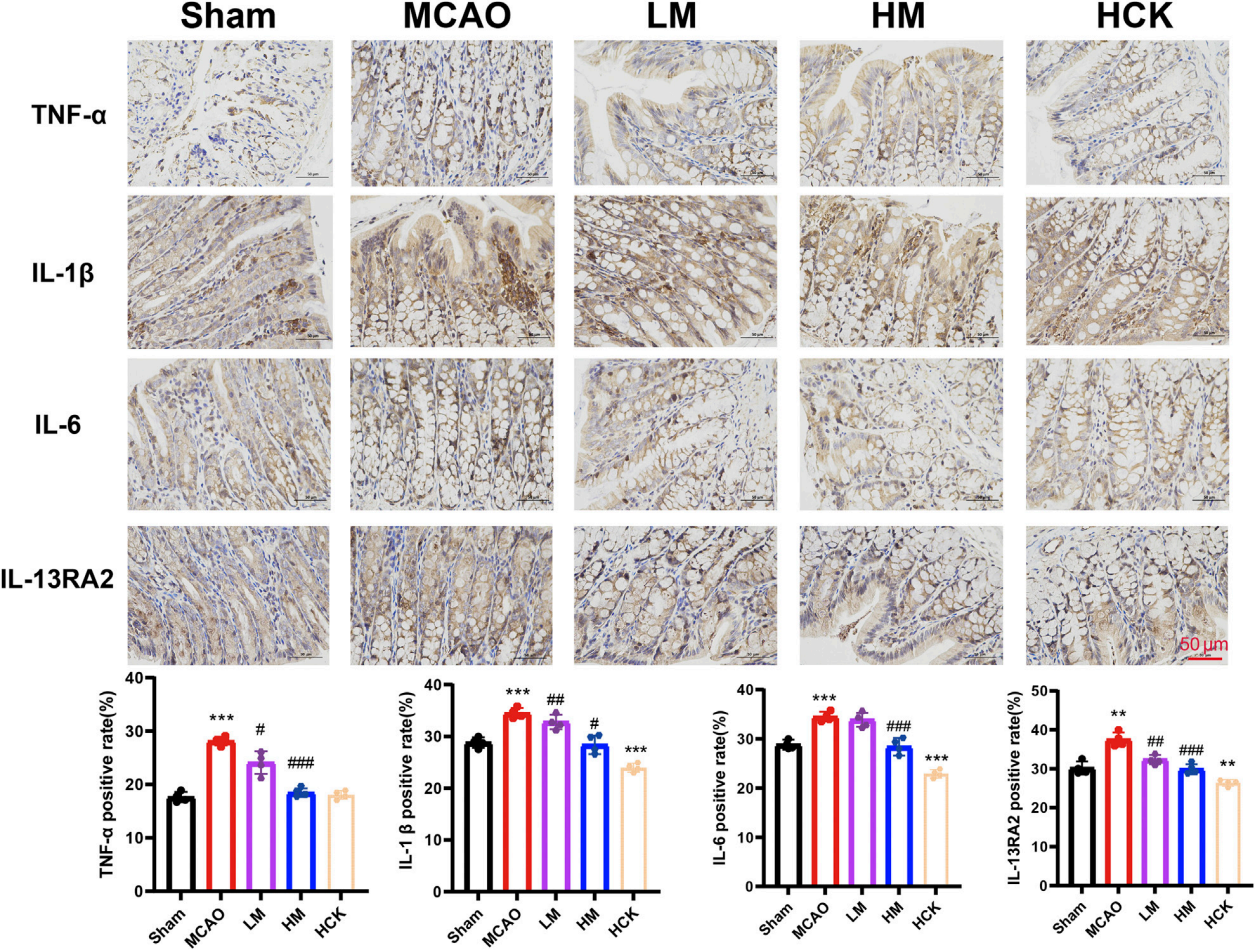
PMP ameliorates MCAO-induced inflammatory response in rats’ colon. Representative immunohistochemical images and statistical maps of inflammatory factors. n = 4. *p < 0.05, **p < 0.01 and ***p < 0.001, MCAO group vs. Sham group; #p < 0.05, ##p < 0.01 and ###p < 0.001, dosing group vs. MCAO group.

Throughout the experiment, significant diarrhea was observed in rats subjected to MCAO modeling rats during the entire experiment, whereas these symptoms were notably attenuated in the HM group. Consequently, experiments to evaluate intestinal injury were conducted. Histopathological examination of the colon (Figure 3A) revealed that I/R injury led to altered villous morphology (white arrow), a reduction in mucosal goblet cells (red arrow), focal immune cell infiltration (black arrows), and thickening of the muscularis mucosa (Supplementary Figure S3). High-dose PMP treatment significantly mitigated colonic tissue damage in the HM group, although a slight degree of inflammatory cell infiltration remained. Of note, the colonic immune-histochemistry results indicated that in the HM group, PMP significantly enhanced the expression of tight junction proteins ZO-1, Occludin, and Claudin, which were compromised by MCAO (Figure 3B). Additionally, the disturbed levels of interleukins TNF-α and IL-6, as well as other inflammatory cytokines, were reversed to varying degree (Figure 4). IL-13Ra2 levels were significantly upregulated by MCAO, which were normalized by PMP. Although limited literature has documented the associations between IL-13RA2 and stroke, it is generally understood that IL-13RA2 can be upregulated by pro-inflammatory factors such as TNF-α and IL-1β (Zhang et al., 2025). The upregulation of IL-13RA2 induced by MCAO may serve as a prognostic indicator. IL-13 is known to promote fibrosis through the stimulation of extracellular matrix production, collagen deposition, and tissue scarring. Initially thought to merely inhibit IL-13 signaling, IL-13RA2 is now recognized as a key regulator of fibrosis, particularly in the gut. Elevated IL-13RA2 levels in tissues indicate its potential role in the progression of inflammatory injury (Brightling et al., 2010). Notably, high-dose PMP nearly reversed the detrimental effects of MCAO on the intestine. These findings imply that PMP may protect colonic tissue from MCAO-induced damage by enhancing colonic barrier proteins and mitigating inflammation.

### 3.3 PMP ameliorated I/R-induced intestinal metabolic disorders in MCAO rats

**FIGURE 5:**
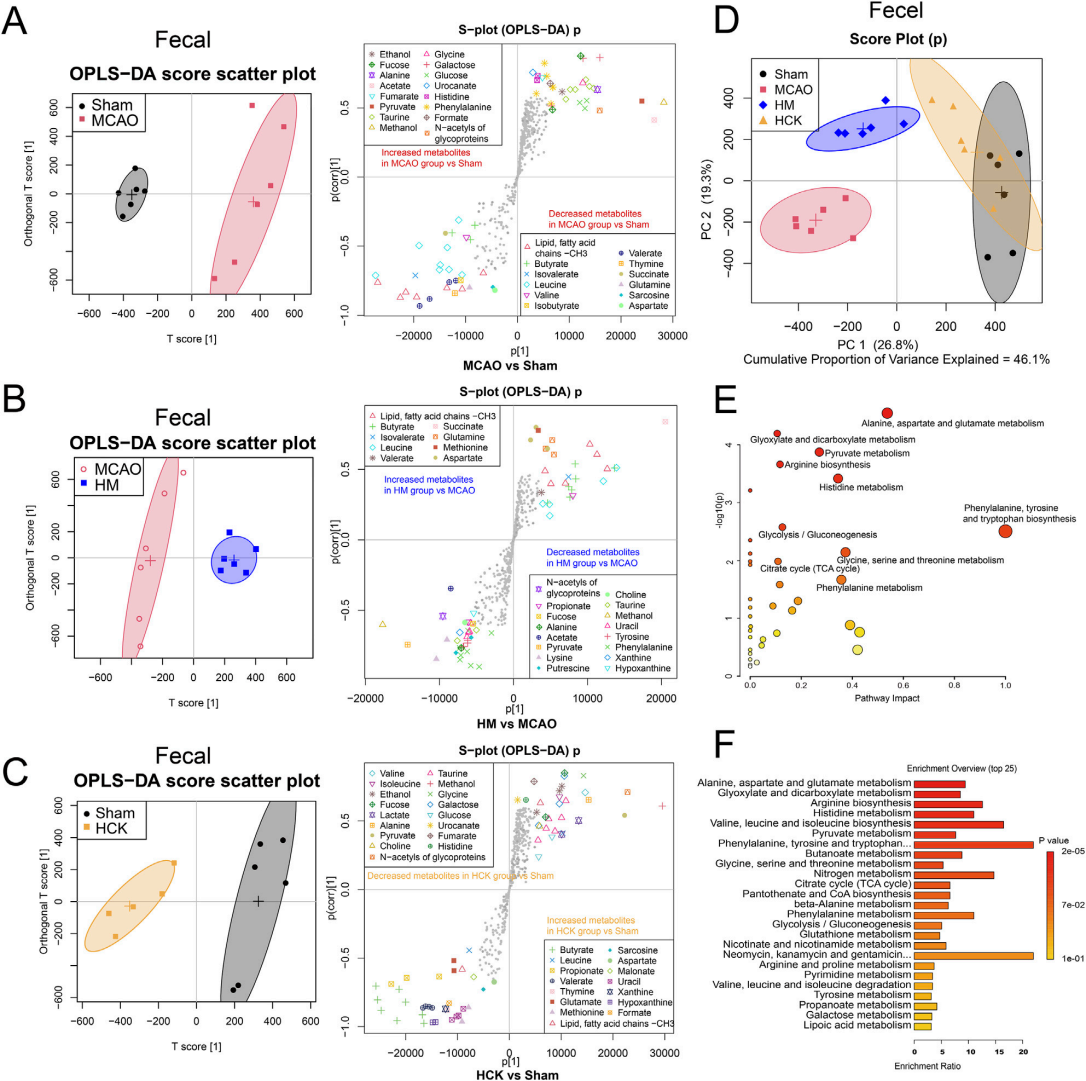
PMP reverses postoperative gut metabolite disturbance in MCAO rats. **(A–C)** OPLS-DA score plots and the associated S-plots between two groups, MCAO vs. Sham, MCAO vs. HM and HCK vs. Sham. **(D)** PCA score plot of four-group comparisons (MACO, Sham, HM and HCK). **(E)** Metabolite set enrichment analysis. **(F)** Enrichment profile of the top 25 metabolic pathways.

Alterations in intestinal microbial metabolites were examined following the induction of the MCAO model and the administration of PMP, utilizing ^1^H based NMR-based metabolomics, as illustrated in Figure 5. In particular, OPLS-DA analysis amplifies differential metabolites, thus revealed alterations in fecal metabolites by MCAO/PMP. A notable reduction in the levels of SCFAs, including butyrate, valerate, and isovalerate, as well as amino acids such as valine, leucine, glutamate, and aspartate, was observed in the MCAO group (Figure 5A). In contrast, in the HM group, the administration of PMP led to a restoration of these specific SCFAs and amino acids (Figure 5B). And in HCK group, PMP also contributes to a significant increase in SCFAs levels, such as propionate, butyrate, and valerate, rats’ intestinal contents (Figure 5C), indicating that PMP promoted the SCFAs production in the metabolic process of the intestinal microorganisms.

The intestinal metabolic profile shifts in OPLS-DA analysis showed that PMP played a role in improving intestinal metabolic disorders induced by MCAO, which was also confirmed in PCA analysis. In PCA score plots (Figure 5D; Supplementary Figure S4), the Sham and MCAO groups were furthest away, indicating that the MCAO-induced I/R injury in brain had a significant impact on the metabolism of intestinal flora. Furthermore, PMP of both high and low dose proved a therapeutic efficacy to I/R-induced intestinal metabolic disorders: the LM/HM groups were located between the Sham and MCAO groups, respectively, suggesting that PMP dose-dependently reversed the intestinal metabolic disturbance. Overlapping distribution between HCK and Sham groups indicated that PMP has little effect on metabolic parameters, which proves its non-toxic nature. These improved indicators mainly contributed to the pharmacological mechanism underlying PMP-induced improved the neurological prognosis. Moreover, the initial metabolic analysis confirms disturbance of gut-brain axis by MCAO surgery, and indicated interactions between PMP and microbiota played an important role in the improved neurological prognosis.

Statistical analysis of representative 31 metabolite profiles was illustrated in Supplementary Figure S5. In total, levels of 44 perturbed metabolites (detailed in Supplementary Table S3) were detected in feces. The levels of butyrate, leucine, propionate, isobutyrate, succinate, and glutamine were significantly decreased, and galactose, tyrosine, phenylalanine, xanthine were significantly increased in MCAO group compared with those in Sham group. PMP treatment reversed the disturbance of the 13 metabolites, shown in the LM and HM groups compared with those in the MCAO group. The metabolites that exhibited alterations in the serum are enumerated in Supplementary Table S4. Pathway enrichment analysis by using MetaboAnalyst 5.0 showed multiple enriched pathways, including nucleotide glucose metabolism, amino acid metabolism (alanine, aspartic acid, glutamic acid, tryptophan, phenylalanine, tyrosine, valine, leucine and isoleucine, glycine, serine and threonine), niacin metabolism, pyruvate metabolism, biosynthesis of pantothenate and coenzyme A, nitrogen metabolism, metabolism of glyoxylate and dicarboxylate, butyric acid metabolism (Figures 5E,F).

### 3.4 PMP regulated homeostasis of intestinal flora in MCAO rats

**FIGURE 6:**
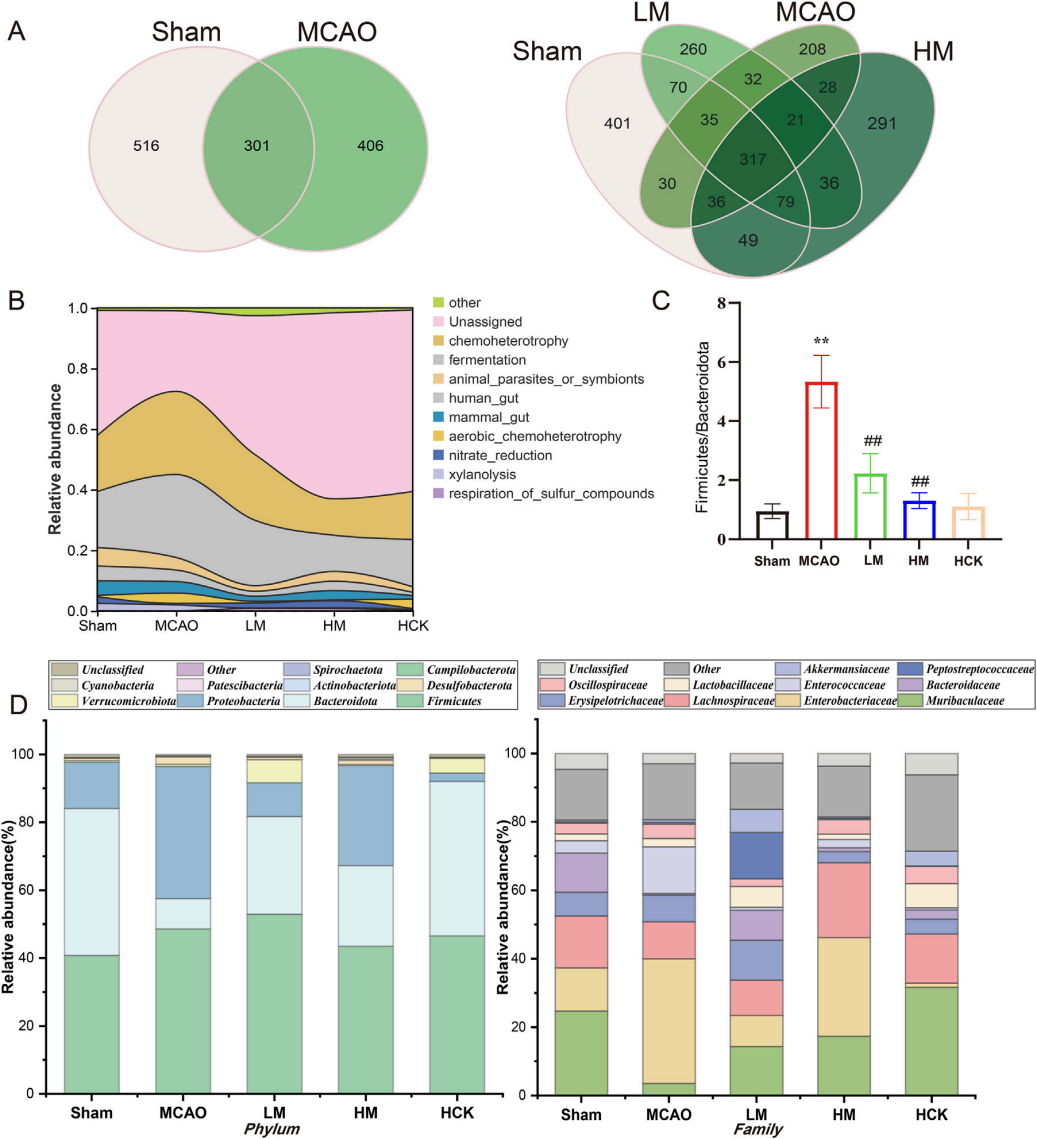
PMP regulated homeostasis of intestinal flora in MCAO rats. **(A)** Venn diagram of the OTUs and overlapping OTUs in different mice groups. **(B)** Functional abundance river maps (based on FAPROTAX, a functional annotated prediction of biogeochemical cycle processes). **(C)** Ratio of the phylum Bacteroidetes in Firmicutes (F/B), n ≥ 3. *p < 0.05, **p < 0.01 and ***p < 0.001, MCAO group vs. Sham group; #p < 0.05, ##p < 0.01 and ###p < 0.001, dosing group vs. MCAO group. **(D)** Relative abundance of gut microbiota at phyla and family levels.

Dilution curves, or rarefaction curves, in 16S rRNA sequencing were used to assess if sequencing depth is enough to capture microbiota diversity. By drawing observed species (OTUs) against sequence count, a significant plateau suggests sufficient coverage, indicating our sequencing data was reliable with enough microbiota diversity (Supplementary Figure S6). To conduct a more detailed examination of the alterations in intestinal microbiota composition, the average operational taxonomic units (OTUs) and the shared OTUs—used to categorize bacteria based on sequence similarity among different taxa—were visualized using Venn diagrams. Approximately 110 OTUs were lost during MCAO modeling. Notably, the number of OTUs exhibited a significant increase following PMP treatment, as evidenced by the larger circle representing the HM group, indicating an improvement in species richness (Figure 6A). Furthermore, the prevalence of bacteria associated with chemoheterotrophy (yellow peak in the MCAO group) and fermentation (grey peak in the MCAO group) functions exhibited a significant increase following MCAO, as demonstrated in Figure 6B. The PMP intervention modulated the functional profiles of the microbiota in the LM and HM groups, rendering them comparable to those observed in the Sham group.

The ratio of abundance between *Firmicutes* and *Bacteroidetes* (Fir/Bac ratio) is a marker of intestinal homeostasis, which also upregulated in gut of MCAO rats, suggesting disorders in intestinal microbiota composition (Figure 6C). Further information is presented in the stacked bar charts (Figure 6D). At the phylum level, healthy rat’s intestinal microbiota predominantly consists of *Firmicutes*, *Bacteroidetes*, and *Proteobacteria*. The abundance of *Bacteroidetes*, known as beneficial bacterium, was significantly decreased in the MCAO group, while the abundance of *Firmicutes* and *Proteobacteria* was significantly elevated. At the family level, the abundance of *Muribaculaceae*, one key intestinal bacterium of *Bacteroidetes*, was significantly downregulated after MCAO modeling, while the abundance of *Enterobacteriaceae*, an opportunistic pathogen, was markedly increased. Surprisingly, PMP ameliorated the disorders in the composition and diversity of intestinal flora: downregulated F/B index and the abundance of opportunistic pathogens, upregulated the abundance of *Muribaculaceae* and *Lactobacillus*. Moreover, the quantifiable data of intestinal microbiota composition from rats in the HCK group, present better and closer tendency to that from rats in the Sham group.

**FIGURE 7:**
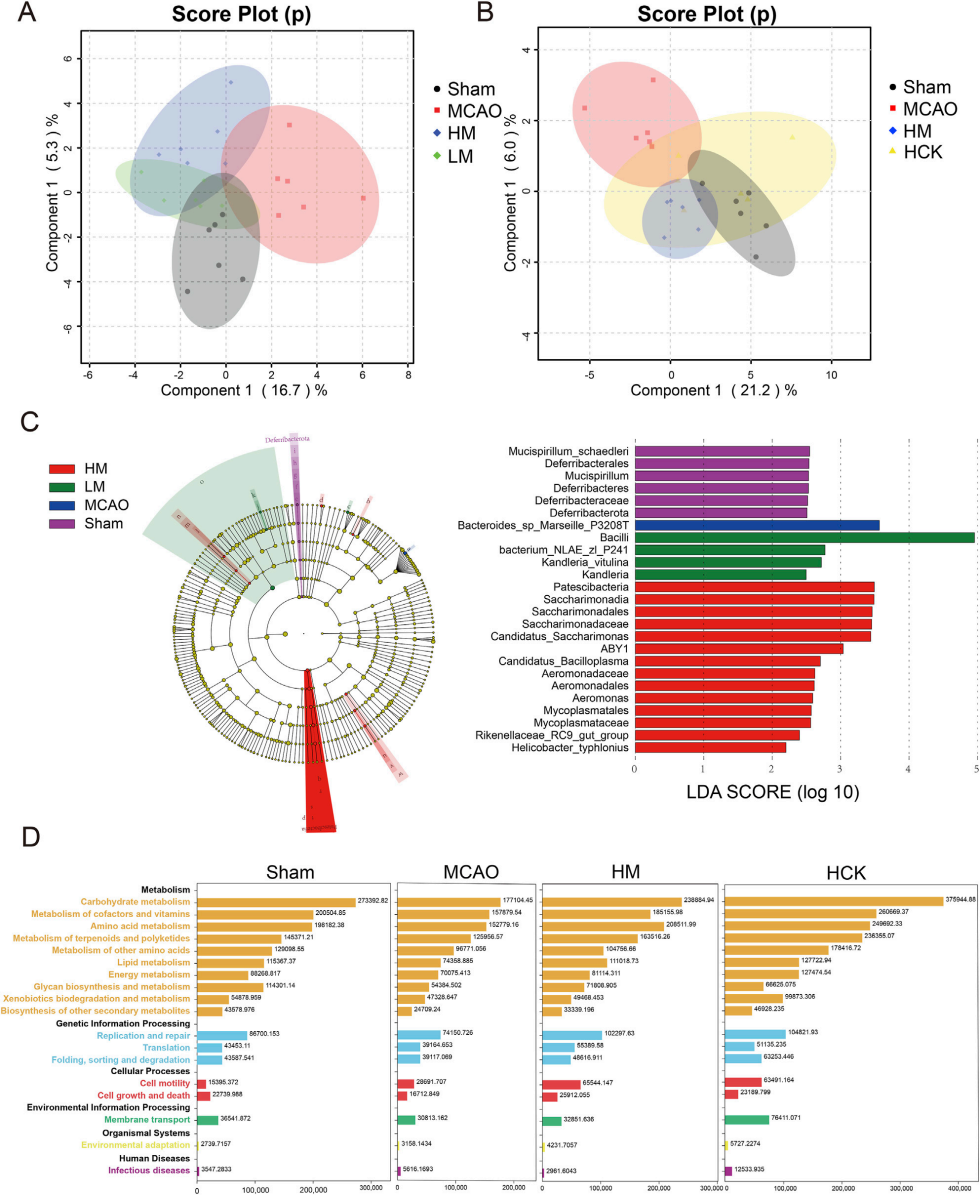
PMP regulated homeostasis of intestinal flora in MCAO rats. **(A,B)** Multivariate statistical analysis was conducted on 16S rRNA sequencing data. Principal Component Analysis (PCA) score plots were generated for comparisons among four groups: **(A)** MACO, Sham, HM, and LM; **(B)** MACO, Sham, HM, and HCK. **(C)** LEfSe multilevel species cladogram of four groups. The brightness of each dot is proportional to the effect size. Only taxa with a significant LDA threshold value >2 are shown. **(D)** Function Distribution Overview map (based on PICRUSt2, software that predicts function abundance based on marker gene sequences).

The PCA score plots of 16S rRNA-seq datasets showed that samples of LM, HM and Sham groups exhibited a degree of clustering on PC1 ranged from about −6 to 0 to some extent, distinguishing them from the MCAO samples (Figure 7A). Similarly, the HCK, HM and Sham groups clustered on PC1 within the range of 0–10, and were distinctly separated from the MCAO samples away from the MCAO samples (Figure 7B). To comprehensively evaluate the taxonomic diversity of the microbial community, we subsequently estimated the species richness of each specimen. The Chao index was employed to quantitatively assess the abundance of microbial communities, revealing a positive correlation between microbial community abundance and species richness. Additionally, the Shannon index and Simpson index were utilized to characterize species diversity, with higher values indicating greater community diversity. Chao, Shannon and Simpson indexes were illustrated in Supplementary Figure S7, and visually decreased in MCAO group. And PMP markedly reversed three indexes. Thus, 16S rRNA-seq statistical analysis signified that PMP recovered the species richness and diversity of microbial communities.

We used the LEfSe algorithm to identify the specific taxa that were variably distributed among the four groups. Three taxa were over-represented, including the phyla *Bifidobacterium*, *Rhodobacteraceae*, and *Lachnospiraceae*, in the HM rats compared with that in the MCAO rats (Figure 7C). Specificly, the abundance of *Blautia glucerasea* and *Bacteroides* in intestinal flora increased significantly after MCAO modeling (Supplementary Figure S8). In addition, PICRUSt2 infers gene family and metabolic pathway abundances from marker gene data, offering insights into gut microbiota functions (Douglas et al., 2020). Overall, in the MCAO group, nearly all metabolic pathways exhibited relatively lower abundance compared to the other groups (Figure 7D; Supplementary Figure S9). Administration of high-dose PMP resulted in an increase in metabolic pathways, including carbohydrate metabolism and amino acid metabolism. This enhancement was universally observed in both the HM and HCK groups. Modeling of MCAO is correlated with a diminished diversity and uniformity of intestinal microbiota species, a reduction in the abundance of beneficial bacterial populations, an increase in the prevalence of pathogenic microflora, and the onset of metabolic disorders. In contrast, the administration of high-dose PMP has demonstrated efficacy in mitigating the dysbiosis and metabolic disturbances induced by MCAO.

**FIGURE 8:**
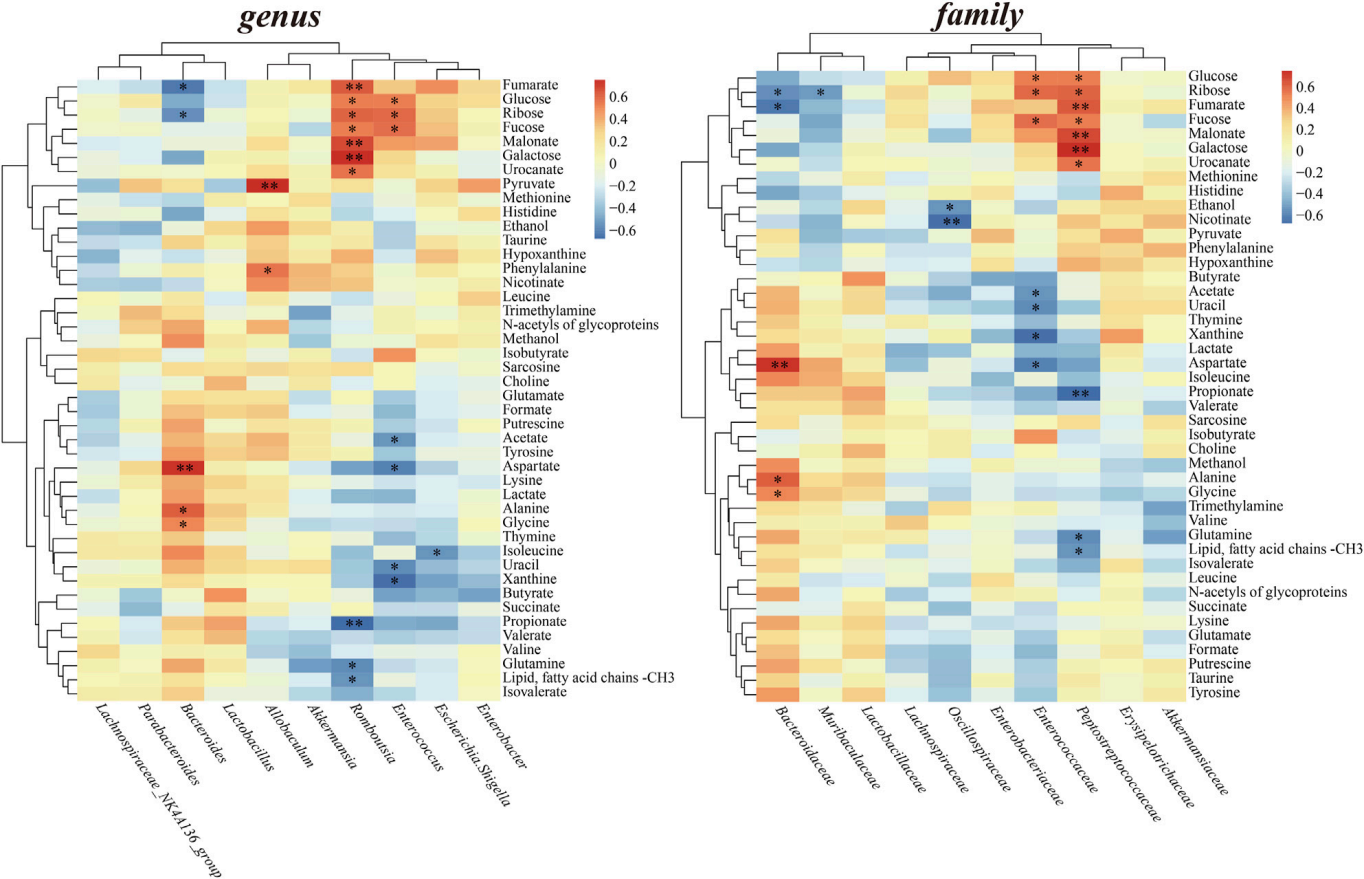
Heatmaps of Spearman’s correlation between perturbed gut microbiota at the genus and family level and altered fecal metabolites.

Spearman’s correlation analysis was performed at both the genus and family levels to investigate the relationships between altered gut microbiota and fecal metabolite levels (Figure 8). *Bacteroide* levels displayed a negative correlation with fumarate and ribose, and showing a positive correlation with aspartate, alanine and glycine. *Allobaculum* levels showed a significant positive correlation with pyruvate, phenylalanine. *Romboutsia* displayed positive correlations with hexose and metabolites involved in Glyoxylate and dicarboxylate metabolism, while negatively correlated with propionate and glutamine. Similarly, *Enterococcus* positively correlated with glucose, ribose and fucose, while showed negative correlations with acetate, aspartate, uracil and xanthine. At family level, *Bacteroidaceae* positively correlated metabolites in amino acid metabolism, such as aspartate, alanine and glycine. *Peptostreptococcaceae* showed positive correlations with all hexose, fumarate, malonate and urocanate; exhibited negative correlations short-chain fatty acid propionate and glutamine.

## 4 Discussion

In this study, the active polysaccharides derived from PM, a well-known nourish TCM, demonstrated high safety and exhibited notable anti-ischemic stroke neuroprotective activity interacting with microbiota. The potential hepatotoxicity of PM has long been a common concern of scholars worldwide and significantly constrains its clinical utilization. Thus, developing and investigating polysaccharides from PM may enhance its therapeutic potential; strengthen its basics in clinical application, while circumventing the associated hepatotoxic risks. Through using ^1^H NMR-based metabolomics combined with 16S rRNA sequenceing, PMP was found upregulating microbiota diversity and the abundance of intestinal probiotics, accelerating SCFA metabolism, moderating amino acid and energy metabolism, thus significantly inhibited the inflammatory cascade to alleviate ischemic brain injury. These results indicated that PMP may represent a novel therapeutic approach for IS in clinic by targeting the intestinal microbiota.

Intestinal dysbiosis initiates in the hyperacute phase (minutes to hours) following a stroke, leading to increased intestinal permeability and the leakage of endothelial cells from the intestine into the bloodstream (El-Hakim et al., 2021). Some studies suggest that SCFAs, particularly butyrate, may play a role in these processes by helping to maintain intestinal permeability and influencing intestinal immunomodulation (Bach Knudsen et al., 2018). Accompanied with the loss of extensive goblet cells, rise of pro-inflammatory factors levels and dysregulation of the tight junction protein (occludin, claudin and ZO-1), decreased levels of SCFAs (butyrate, valerate, and isovalerate) in MCAO group indicated a positive correlation between SCFAs and intestinal injury post-infarction. Gut-derived SCFAs also directly or indirectly impact immunity at extraintestinal sites such as the liver, lungs, reproductive tract, and brain (Mann et al., 2024). They could influence leukocyte movement from the gut to other tissues, and affect brain microglia function and the blood-brain barrier (Erny et al., 2015). Supraphysiological intranasal treatment with sodium butyrate 1 h after MCAO has been shown to reduce infarct size significantly while improving neurologic function at 24 and 72 h (Zhou et al., 2021). Butyrate is utilized by colon cells for energy production while stabilizing hypoxia-inducible factors to maintain an anaerobic environment within the gut. It also helps maintain integrity of the intestinal barrier by regulating Claudin-1 expression and limiting pro-inflammatory cytokines (Singh et al., 2022). Interestingly, PMP maintained intestinal barrier function and upregulated levels of intestinal SCFAs (butyrate, propionate), showing a prebiotic characteristic to a certain degree.

The colon serves as the primary production site in human body, with most being produced through the fermentation of undigested and absorbed carbohydrates by anaerobic bacteria (Jing et al., 2021). Based on PICRUSt2 predictive analysis of sequencing results carbohydrate metabolism is predicted to be significantly upregulated in the PMP group compared to that in the MCAO group (Figure 7D). Human alimentary enzymes are not able to digest most plant polysaccharides. Instead, these polysaccharides are metabolized by microbes which generate SCFAs (Holscher, 2017). In laboratory, polysaccharides from Seeds of Plantago asiatica L (Li et al., 2019), Ganoderma atrum (Zhu et al., 2016), Aloe (Hamman, 2008), Codonopsis (Cao et al., 2022) and Poria cocos (Duan et al., 2023) were all found involved in the production and secretion of SCFAs, by metabolizing in the colon, stimulating the growth of probiotics and improving of intestinal function, meanwhile exhibited various pharmacological activities. Based on structural speculation, we might attribute the abilities, offering short-chain fatty acids, to the common carbon skeleton of polysaccharides and carbohydrates, and of course, to the participation of gut flora.

SCFAs-producing bacteria mostly reported were *Lactobacillus, Bifidobacterium and Clostridium* (Cheng et al., 2022). For instance, certain *Clostridium* species of the phylum *Firmicutes* (*C. acetobutylicum*, *C. butyricum*, *C. pasteurianum*, *C. perfringens*), along with *Butyrivibrio fibrisolvens* and *Fusobacterium* *nucleatum*, are involved in butyric fermentation (Louis et al., 2014). A cocktail of SCFA-producing probiotics, *B. longum*, *C. symbiosum*, *F. prausnitzii*, and *L. fermentum* and inulin (a kind of prebiotic) transplanted into aged mice 3 days after stroke, could ameliorate stroke-induced intestinal damage by stimulating epithelial cells to produce mucin, which supports gut barrier function (Van der Post et al., 2019). More, this cocktail appeared sufficient to improve neurological deficits, motor function, grip strength and depressive phenotypes when compared with mice treated with inulin alone, SCFA-producing alone, or vehicle (Lee et al., 2020). In our study, levels of *Bifidobacterium*, *Muribaculaceae*, *Lactobacillus* strengthened by high-dosed PMP supplementation. Among them, *Bifidobacterium* has been found relevant to long-term rehabilitation of mice with cerebral ischemia (Rahman et al., 2024). The gut microbiota is predominantly composed of the phyla *Bacteroidetes* and *Firmicutes*, playing a vital role in metabolic processes and immune function. Within the order *Bacteroidetes*, the family *Muribaculaceae* is notable for its ability to produce SCFAs from both endogenous sources, such as mucin glycans, and exogenous polysaccharides, considered a promising “next generation probiotic” (Ormerod et al., 2016). Moreover, especially in metabolizing polysaccharides, this bacterial family was found engaged in a cross-feeding relationship with probiotic genera, including *Bifidobacterium* and *Lactobacillus* (Zhu et al., 2024). To *Bifidobacterium*, carbohydrates are always the main class of nutrients for its growth. The beneficial effects of the polysaccharides diet have been linked to an increased prevalence of *Muribaculaceae*, suggesting its potential in treating IS. TCM polysaccharides are highly likely to become a better source of nutrition, like SCFAs, for intestinal flora.

Furthermore, correlation analysis demonstrated propionate was negatively correlated with the levels of family *Peptostreptococcaceae* and the *genus Romboutsia* (Figure 8). Members of the *Peptostreptococcaceae* family/*Romboutsia* genus belonging to the order *Clostridiales*, phylum *Firmicutes*. High *Peptostreptococcaceae* abundance was considered correlated with lower microbial diversity of the endogenous microbiota (Zaccaria et al., 2023). *Romboutsia* is considered a noxious bacteria negatively impacting gut microbiota and host metabolism, harmful to SCFA production and probiotic colonization (Bai et al., 2023). Like *Enterobacteriaceae*, an opportunistic pathogen, *Romboutsia* might also be a risk factor or a sign of poor prognosis in IS correlated with SCFAs. Of course, the relationships between these specific bacteria and SCFAs production in case of IS still needs to be further confirmed and clarified.

In addition, there is evidence that gut microbiota directly regulates host regulation of energy balance (Heiss and Olofsson, 2018). Besides intaking carbohydrates, amino acid metabolism could be another way to offer SCFAs. Amino acids can produce butyrate through the glutamate pathway under the help of specific producing microorganisms (Singh et al., 2022). Also, exogenous amino acids supplementation could eliminate differences in inflammation, gut microbiota, and overall disease severity in mice contribute to the inflammatory bowel disease (IBD). (Andrew et al., 2018). In our study, *Bifidobacterium* and *Lactobacillus* have been significantly increased with the enhanced amino acid metabolism in HM group. *Bifidobacterium* has been shown to produce essential nutrients such as alanine, valine, aspartic acid and threonine (Wang et al., 2018). Meanwhile, the family *Bacteroidaceae* and *the genus Bacteroides* both displayed positive correlations with amino acids, such as aspartate, alanine and glycine. Although our data do not suggest *Bacteroides* levels were related to the production of SCFAs, indirect evidence related to amino acid metabolism can infer the relevance of *Bacteroides* to SCFAs which have been widely reported. The composition of intestinal flora played an important role in PMP increasing short-chain fatty acids and regulating amino acid levels, thus bringing overall long-term rehabilitation.

## 5 Conclusion

PMP significantly alleviate cerebral I/R injury by modulating the gut-brain axis. By promoting beneficial gut microbiota (particularly *Bifidobacterium*, *Muribaculaceae*, and *Lactobacillus*), and enhancing SCFA production, PMP preserves intestinal integrity and lowers inflammatory responses. These effects collectively reduce infarction volume and improve neurological function in MCAO/R rats. Hence, PMP holds promise as a prebiotic-based therapeutic intervention for preventing and managing IS, underscoring its potential in clinical applications. Our findings have demonstrated the interactions between TCM polysaccharides and the gut microbiota, including their effects on the host immune system and intestinal bacteria. Moreover, perturbations in the gut-brain axis were confirmed in IS, suggesting that the gut microbiota represents a promising therapeutic target for the prevention and treatment of IS.

## Data Availability

The metabolomics data presented in this study have been deposited in the ZENODO repository and are publicly accessible at https://zenodo.org/records/15374056. Further inquiries can be directed to the corresponding authors.
