# Correction: Polygoni Multiflori Radix Praeparata polysaccharides enhance gut health and mitigate ischemic stroke by regulating SCFA and amino acid metabolism in gut microbiota

**DOI:** 10.3389/fphar.2025.1636607

**Published:** 2025-06-16

**Authors:** Lingyu Ruan, Zhennan Wang, Mengyun Zheng, Qi Zheng, Qing Qing, Hongyan Lin, Yuheng Tao, Liqun Wang, Junsong Wang, Wenhao Ge

**Affiliations:** ^1^ School of Pharmacy and School of Biological and Food Engineering, Changzhou University, Changzhou, China; ^2^ School of Pharmacy, Modern Industrial College of Traditional Chinese Medicine and Health, Lishui University, Lishui, China; ^3^ Center of Molecular Metabolism, Nanjing University of Science and Technology, Nanjing, China; ^4^ The Second People’s Hospital of Changzhou, The Third Affiliated Hospital of Nanjing Medical University, Changzhou, China; ^5^ Changzhou Medical Center, Nanjing Medical University, Changzhou, China

**Keywords:** polygoni multiflori radix praeparata, polysaccharides, acute ischemic stroke, gut microbiota, metabolomics

There was an error in Figure 3 as published. The authors inadvertently substituted a 40× image from the Sham group with a 40× image from the HCK group in Figure 3A. Because intestinal histology differs markedly between groups (fold architecture, mucosal arrangement, etc.), the mismatch was readily apparent. The incorrect original 40× image of the Sham group was an enlarged image of the HCK 20× image. The authors have now retrieved the correct 40× micrographs for both the Sham and HCK groups from the raw data and have reconstructed Figure 3A accordingly. The corrected Figure 3 appears below.

**FIGURE 3:**
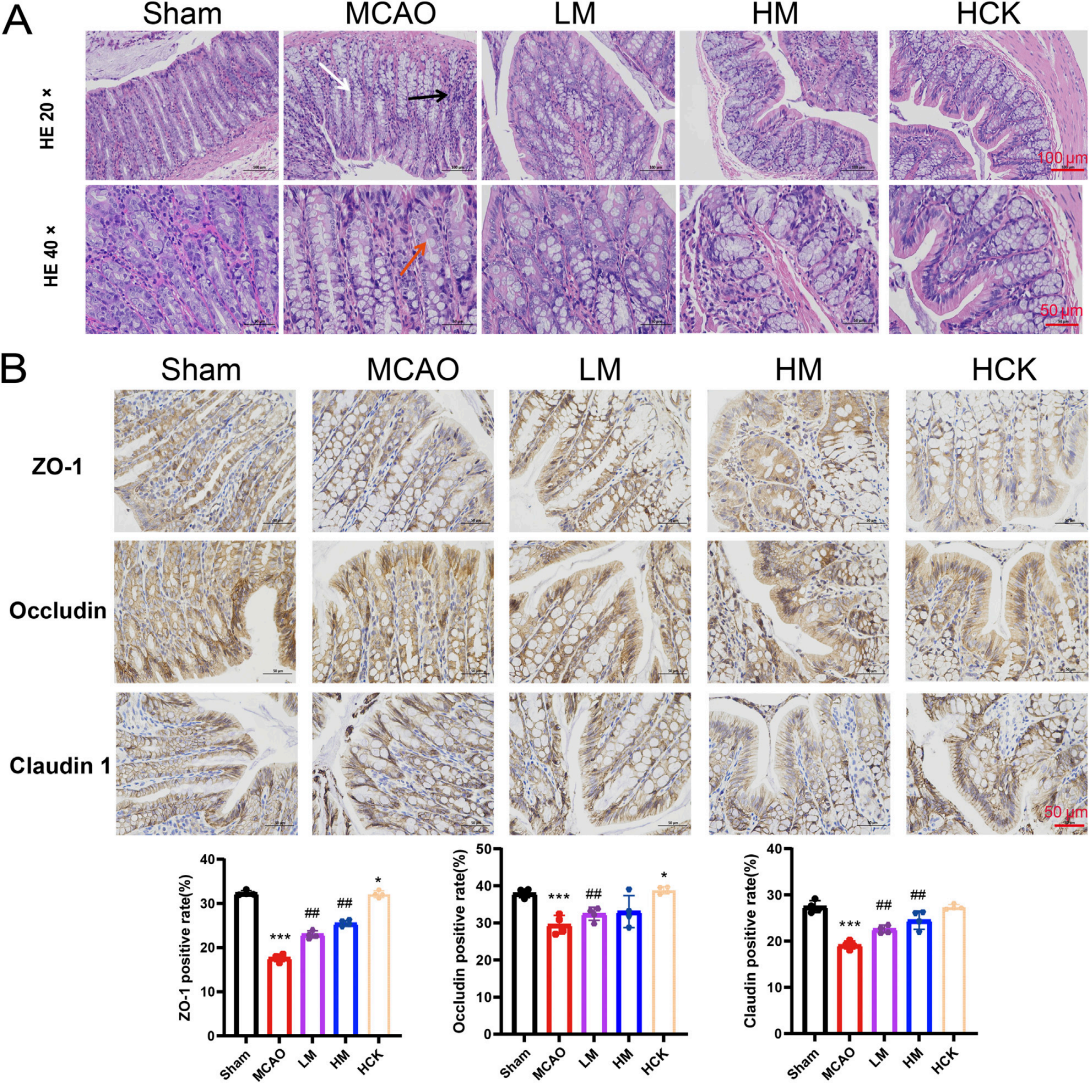
PMP has an ameliorating effect on colon injury in MCAO rats. **(A)** Representative micrographs of H&E staining in the colon. **(B)** Representative immunohistochemical images and statistical maps of tight junction protein. n = 4. *p < 0.05, **p < 0.01 and ***p < 0.001, MCAO group vs. Sham group; #p < 0.05, ##p < 0.01 and ###p < 0.001, dosing group vs. MCAO group.

There was an error in Figure 4 as published. Previously, in Figure 4B the scale bar was positioned outside the image. This has now been adjusted to ensure a consistent style. The corrected Figure 4 appears below.

**FIGURE 4:**
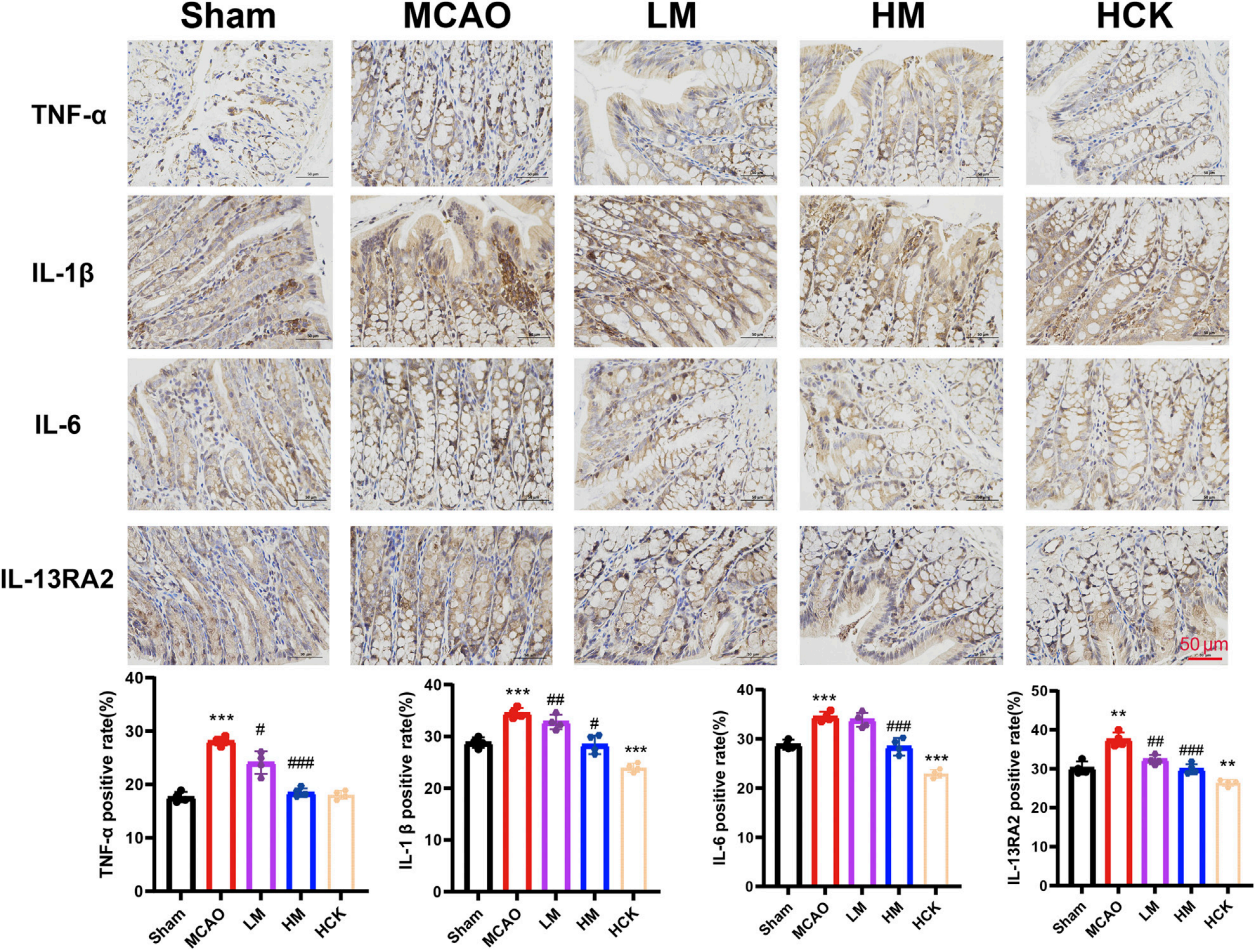
PMP ameliorates MCAO-induced inflammatory response in rats' colon. Representative immunohistochemical images and statistical maps of inflammatory factors. n = 4. *p < 0.05, **p < 0.01 and ***p < 0.001, MCAO group vs. Sham group; #p < 0.05, ##p < 0.01 and ###p < 0.001, dosing group vs. MCAO group.

The original version of this article has been updated.

