# Supplementary material for: Polygoni Multiflori Radix Praeparata polysaccharides enhance gut health and mitigate ischemic stroke by regulating SCFA and amino acid metabolism in gut microbiota
Source: Front Pharmacol. 2025 May 22;16:1580055. doi: 10.3389/fphar.2025.1580055 (PMC12138258; doi:10.3389/fphar.2025.1580055)
Supplement: Supplementary file 1 [file DataSheet1.docx]

Supplementary Material

# Supplementary Figures and Tables

## Supplementary Figures

##
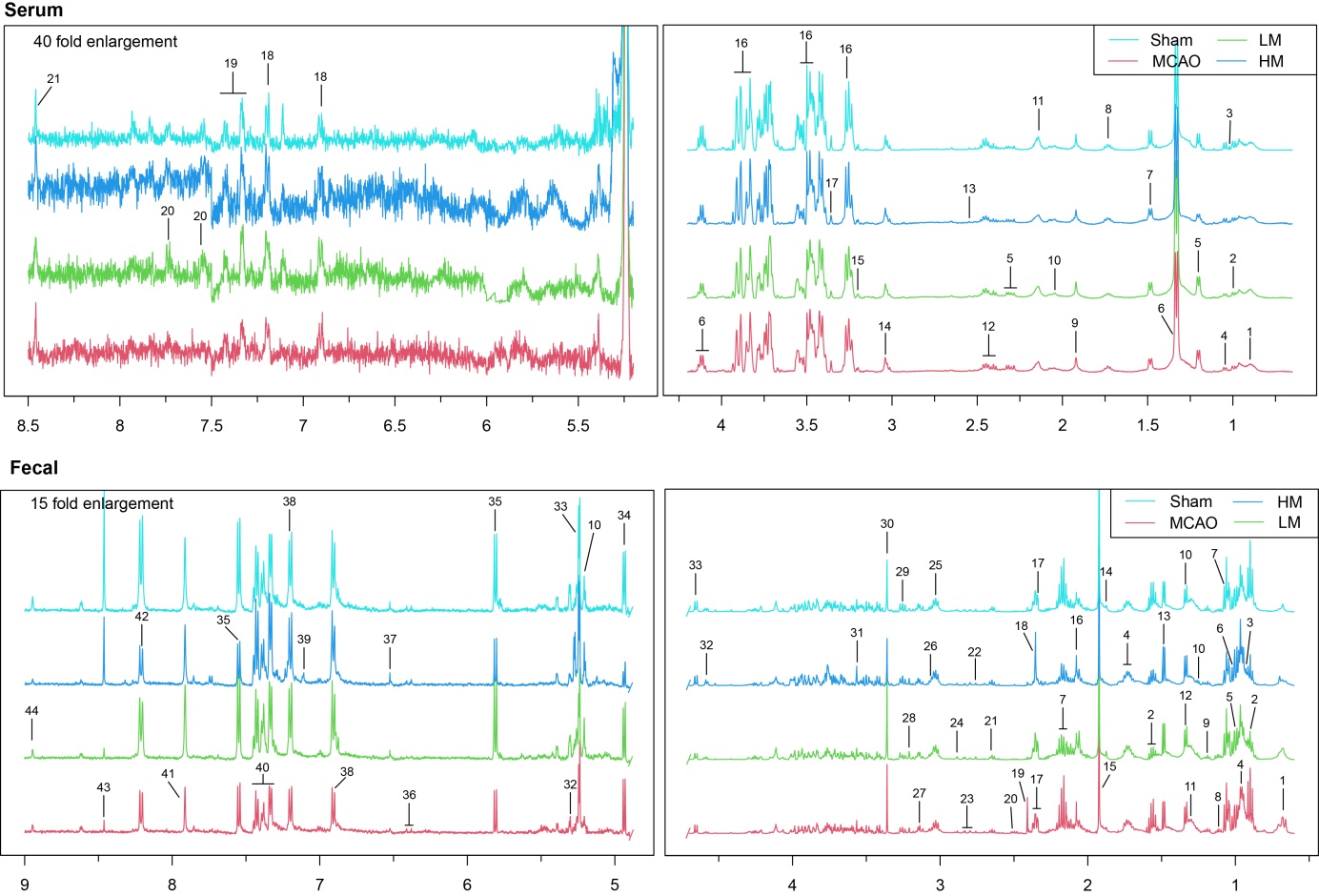


## Supplementary Figure 1. ^1^H NMR spectra of Fecal and serum.


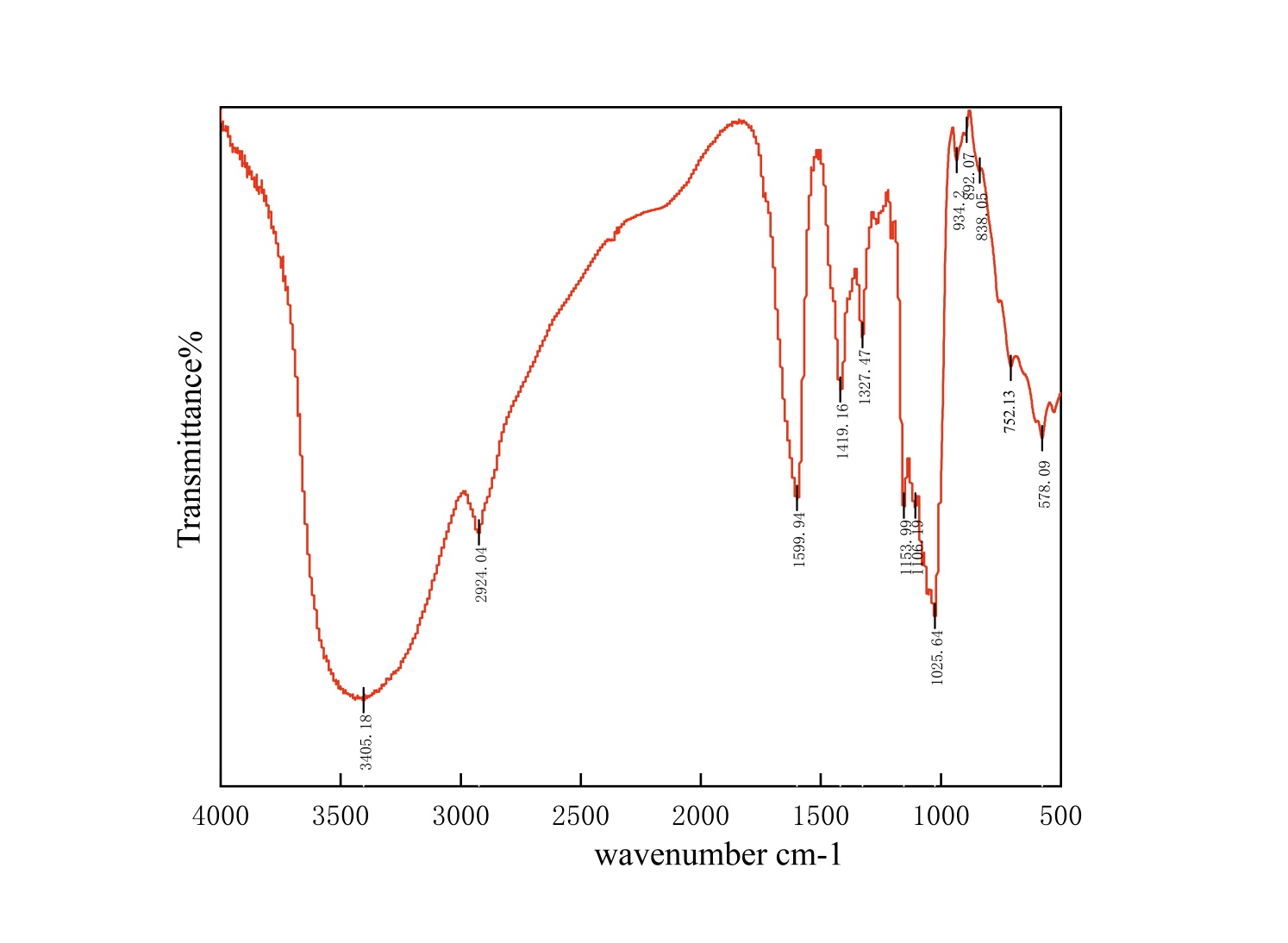


**Supplementary Figure 2.** The fourier transform infrared spectra of PMP.


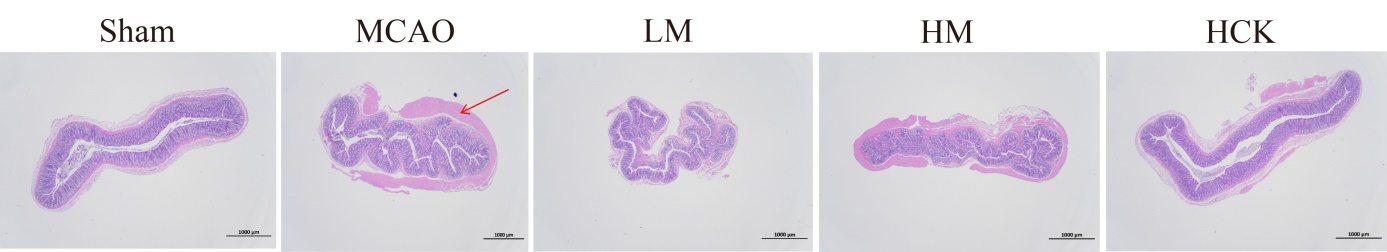


**Supplementary Figure 3.** Pathological section of colon.


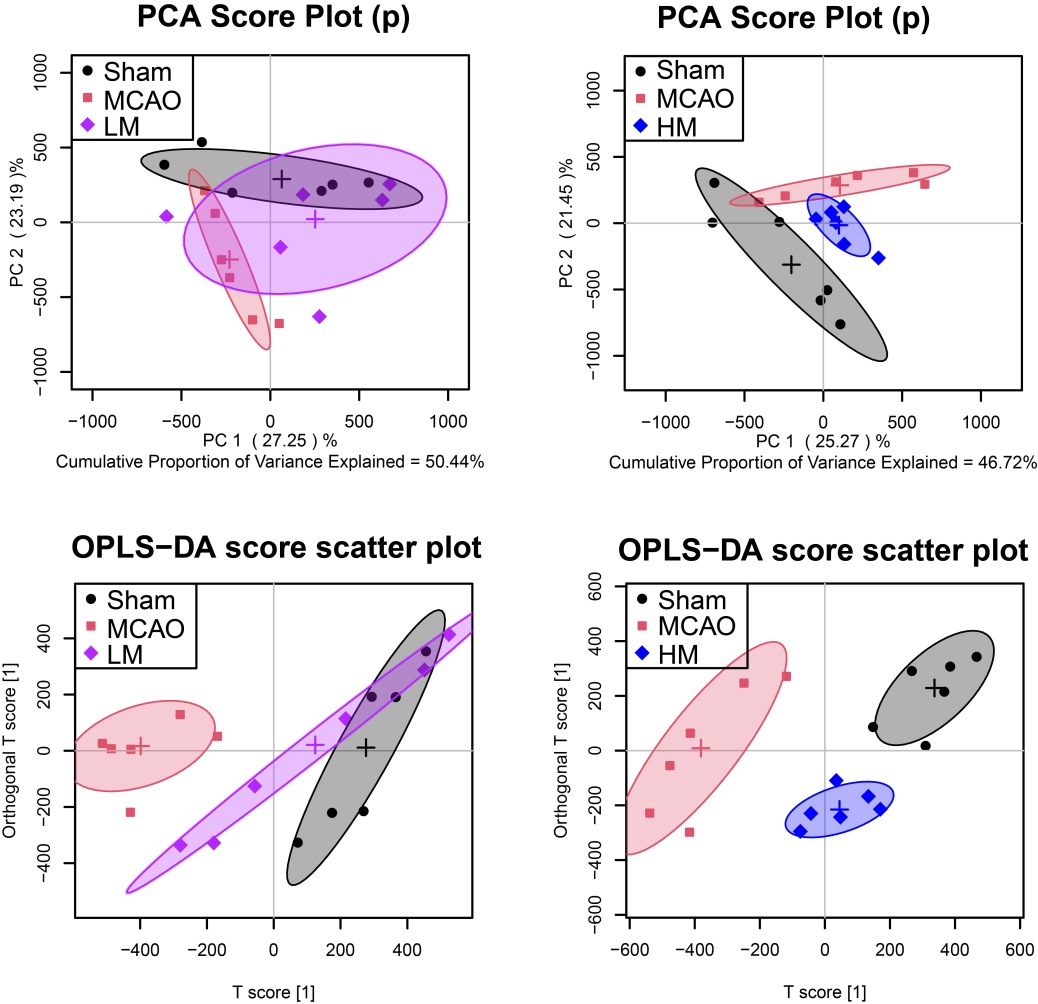


**Supplementary Figure 4.** The PCA and OPLS-DA analysis diagrams based on ^1^H NMR data of rats’ fecal metabolites.


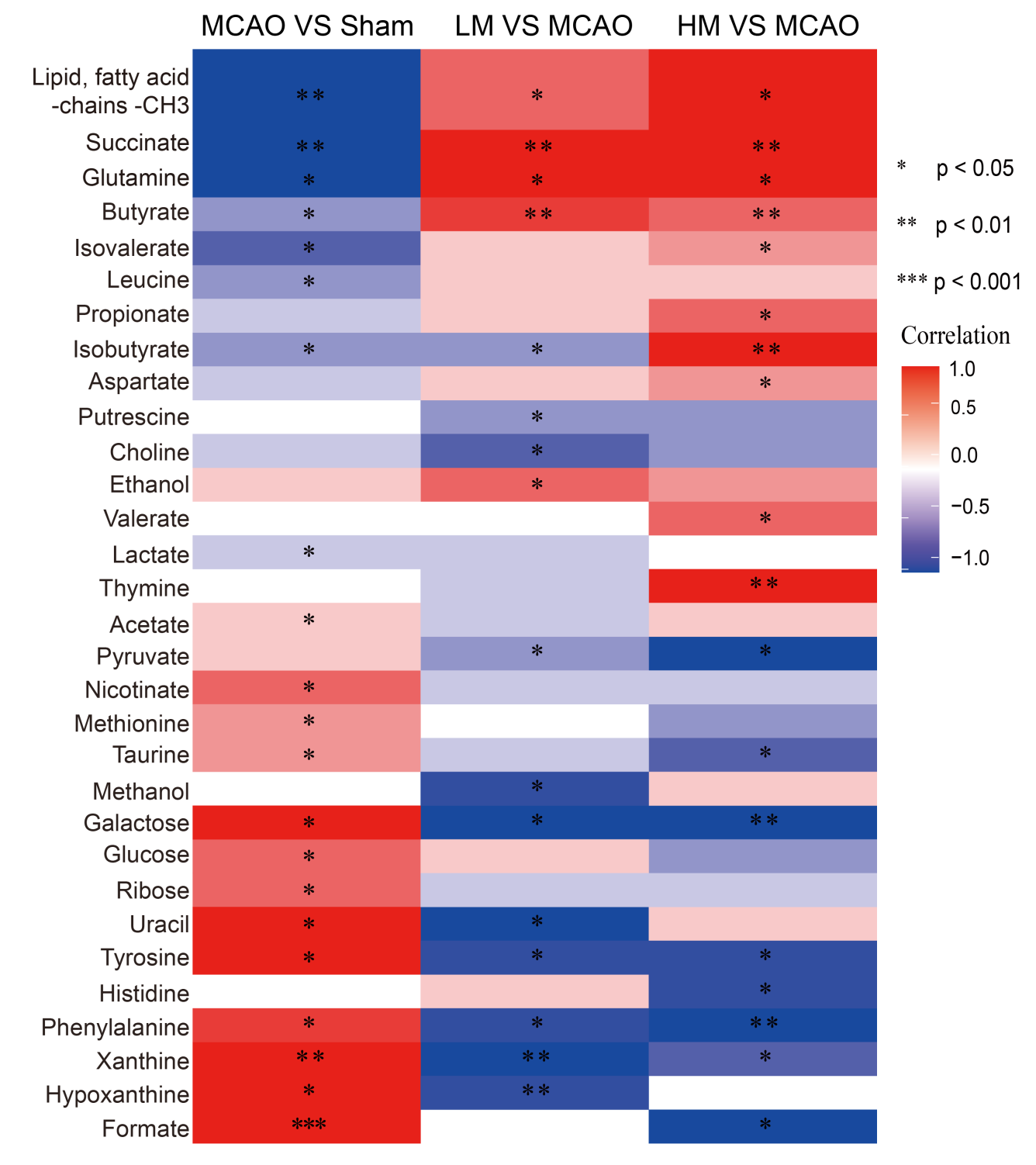


**Supplementary Figure 5.** Heat map of fecal metabolites (analytical method log_2_(FC)). Red indicates an increase in concentration, while blue indicates a decrease. * p < 0.05, ** p < 0.01 and *** p < 0.001 (B)


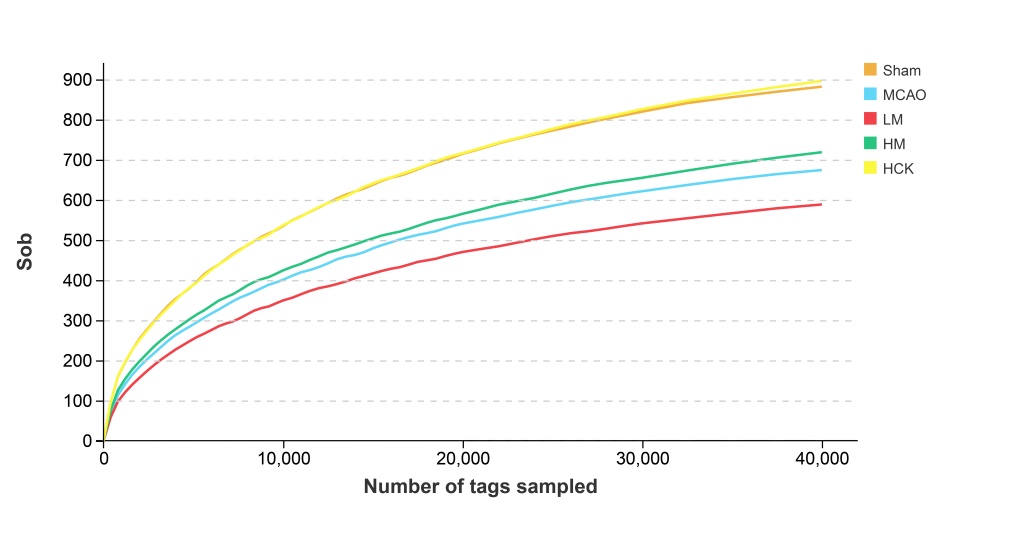


**Supplementary Figure 6.** Fecal 16S RNA dilution curve.


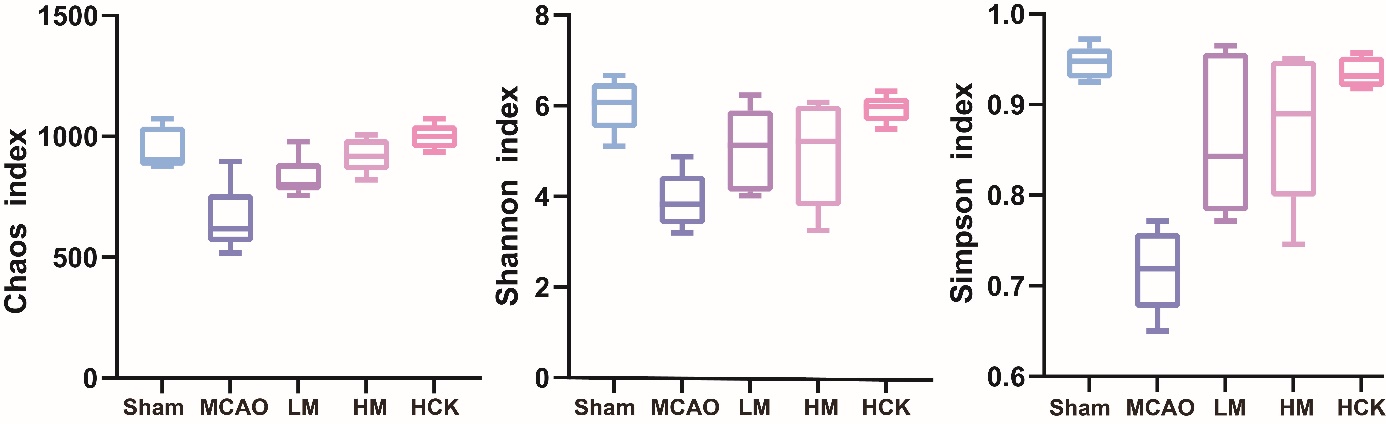


**Supplementary Figure 7.** Chao, Shannon, Simpson box plots. The influence of genera on unique gut microbiota in Sham mice, MCAO mice, LM mice and HM mice was assessed by LEFSE analysis (n=3).


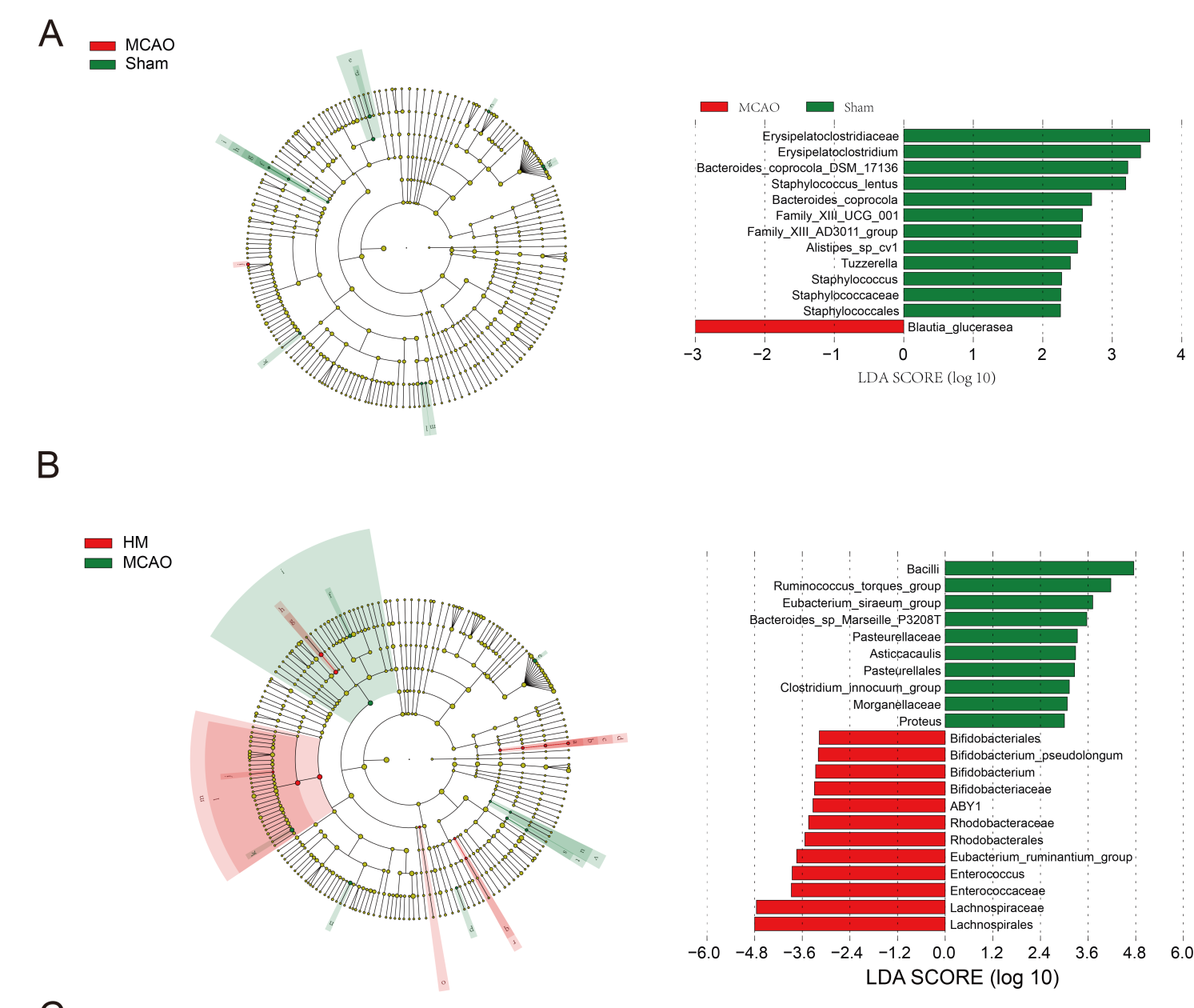


**Supplementary Figure 8.** LEfSe multilevel species cladogram between MCAO and Sham group (A), MCAO and HM group (B). The brightness of each dot is proportional to the effect size. Only taxa with a significant LDA threshold value >2 are shown.


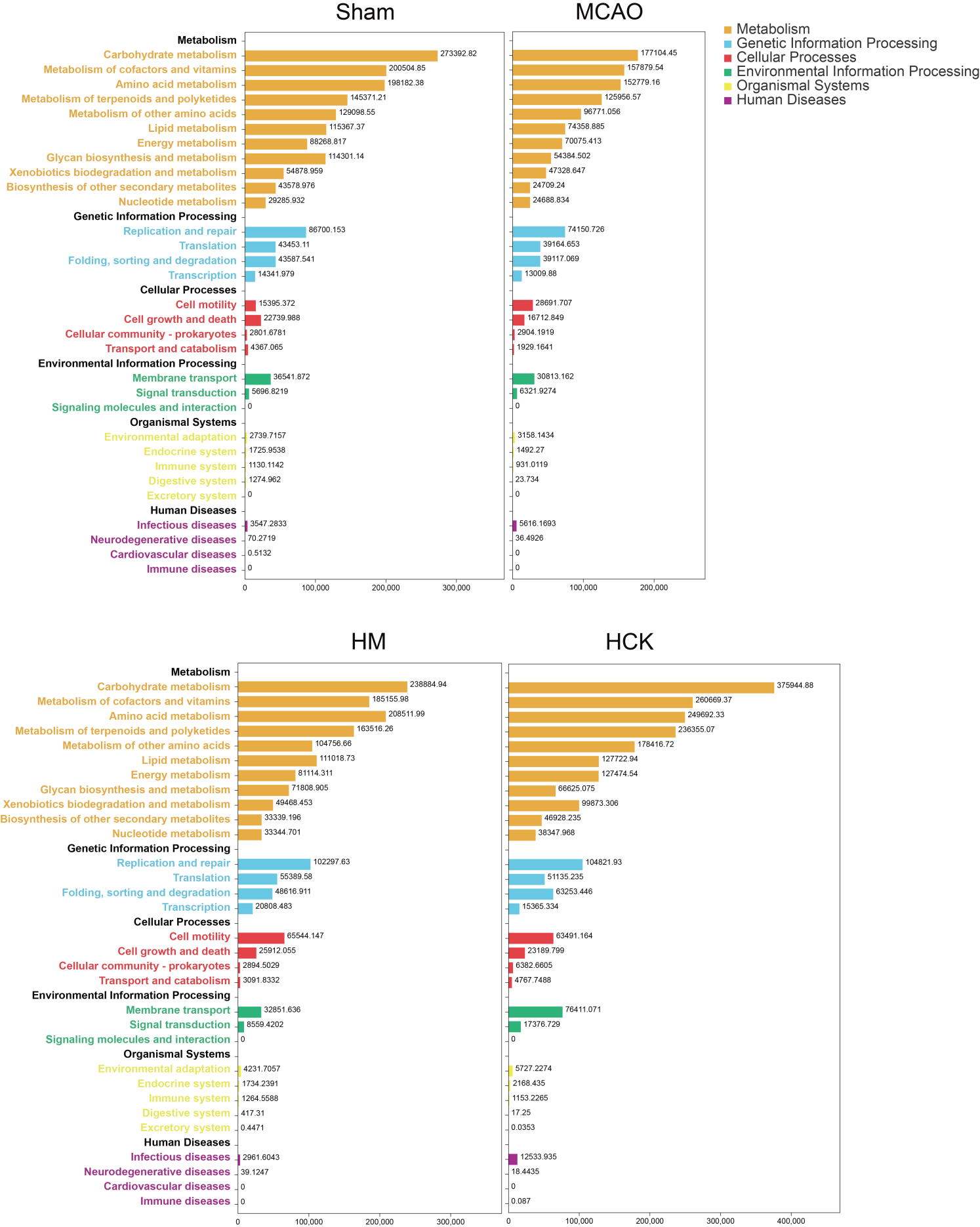


**Supplementary Figure 9.** Complete Function Distribution Overview map (based on PICRUSt2, software that predicts function abundance based on marker gene sequences).

## Supplementary tables

Table.S1 Serum metabolite identification

| No. | Metabolites | Chemical shift | Chemical shift | Chemical shift | | |
| --- | --- | --- | --- | --- | --- | --- |
| 1 | Lipid, fatty acid chains -CH3 | 0.85-0.93 |  | |  |  |
| 2 | Leucine | 0.93-0.975 |  | |  |  |
| 3 | Valine | 0.98-1.01 | 1.03-1.06 | |  |  |
| 4 | Isoleucine | 1.01-1.03 |  | |  |  |
| 5 | 3-Hydroxybutyrate | 1.19-1.22 | 2.275-2.34 | |  |  |
| 6 | Lactate | 1.31-1.35 | 4.09-4.14 | |  |  |
| 7 | Alanine | 1.47-1.50 |  | |  |  |
| 8 | Lysine | 1.70-1.76 |  | |  |  |
| 9 | Acetate | 1.91-1.93 |  | |  |  |
| 10 | N-acetyls of glycoproteins | 2.03-2.09 |  | |  |  |
| 11 | Lipid, fatty acid chains -CH_2_ | 2.11-2.19 |  | |  |  |
| 12 | Glutamine | 2.44-2.47 |  | |  |  |
| 13 | Malate | 2.64-2.66 |  | |  |  |
| 14 | Creatine | 3.03-3.05 | 3.93-3.94 | |  |  |
| 15 | Choline | 3.19-3.205 |  | |  |  |
| 16 | Glucose | 3.23-3.28 | 3.40-3.51 | | 3.70-3.80 |  |
| 17 | Methanol | 3.35-3.365 |  | |  |  |
| 18 | Tyrosine | 6.89-6.92 | 7.18-7.20 | |  |  |
| 19 | Phenylalanine | 7.32-7.45 |  | |  |  |
| 20 | Tryptophan | 7.53-7.56 | 7.72-7.75 | |  |  |
| 21 | Formate | 8.45-8.47 |  | |  |  |

Table.S2 Fecel metabolite identification

| No. | Metabolite | Chemical shift | Chemical shift |
| --- | --- | --- | --- |
| 1 | Lipid, fatty acid chains -CH_3_ | 0.60-0.75 |  |
| 2 | Butyrate | 0.87-0.92 | 1.52-1.595 |
| 3 | Isovalerate | 0.92-0.94 |  |
| 4 | Leucine | 0.94-0.98 | 1.70-1.76 |
| 5 | Valine | 0.98-1.01 |  |
| 6 | Isoleucine | 1.015-1.03 |  |
| 7 | Propionate | 1.055-1.09 | 2.155-2.20 |
| 8 | Isobutyrate | 1.09-1.11 |  |
| 9 | Ethanol | 1.165-1.21 |  |
| 10 | Fucose | 1.24-1.265 | 5.20-5.22 |
| 11 | Valerate | 1.27-1.32 |  |
| 12 | Lactate | 1.32-1.355 |  |
| 13 | Alanine | 1.47-1.50 |  |
| 14 | Thymine | 1.865-1.88 |  |
| 15 | Acetate | 1.915-1.94 |  |
| 16 | N-acetyls of glycoproteins | 2.07-2.085 |  |
| 17 | Glutamate | 2.33-2.35 | 2.36-2.38 |
| 18 | Pyruvate | 2.35-2.36 |  |
| 19 | Succinate | 2.405-2.42 |  |
| 20 | Glutamine | 2.475-2.525 |  |
| 21 | Methionine | 2.63-2.685 |  |
| 22 | Sarcosine | 2.75-2.765 |  |
| 23 | Aspartate | 2.785-2.85 |  |
| 24 | Trimethylamine | 2.875-2.89 |  |
| 25 | Lysine | 3.01-3.05 |  |
| 26 | Putrescine | 3.05-3.08 |  |
| 27 | Malonate | 3.13-3.145 |  |
| 28 | Choline | 3.20-3.22 |  |
| 29 | Taurine | 3.23-3.28 | 3.40-3.45 |
| 30 | Methanol | 3.35-3.37 |  |
| 31 | Glycine | 3.56-3.575 |  |
| 32 | Galactose | 4.58-4.61 | 5.265-5.285 |
| 33 | Glucose | 4.64-4.67 | 5.23-5.25 |
| 34 | Ribose | 4.925-4.95 |  |
| 35 | Uracil | 5.795-5.825 | 7.53-7.57 |
| 36 | Urocanate | 6.37-6.42 |  |
| 37 | Fumarate | 6.52-6.53 |  |
| 38 | Tyrosine | 6.895-6.93 | 7.185-7.22 |
| 39 | Histidine | 7.10-7.125 | 7.845-7.86 |
| 40 | Phenylalanine | 7.32-7.46 |  |
| 41 | Xanthine | 7.90-7.93 |  |
| 42 | Hypoxanthine | 8.19-8.235 |  |
| 43 | Formate | 8.455-8.47 |  |
| 44 | Nicotinate | 8.94-8.955 |  |

Table.S3 Comparison of fecal differential metabolites

| No. | Metabolite | MCAO VS Sham | | LM VS MCAO | | HM VS MCAO | |
| --- | --- | --- | --- | --- | --- | --- | --- |
|  |  | log_2_（FC） | *P* value | log_2_（FC） | *P* value | log_2_（FC） | *P* value |
| 1 | Lipid, fatty acid chains -CH3 | -1.65 | ** | 0.55 | * | 0.93 | * |
| 2 | Butyrate | -0.46 | * | 0.73 | ** | 0.53 | ** |
| 3 | Isovalerate | -0.50 | * | 0.15 |  | 0.47 | * |
| 4 | Leucine | -0.43 | * | 0.1 |  | 0.1 |  |
| 5 | Valine | -0.23 |  | -0.14 |  | -0.19 |  |
| 6 | Isoleucine | -0.26 |  | -0.28 |  | -0.11 |  |
| 7 | Propionate | -0.30 |  | 0.3 |  | 0.56 | * |
| 8 | Isobutyrate | -0.48 | * | -0.46 | * | 1.04 | ** |
| 9 | Ethanol | 0.25 |  | 0.53 | * | 0.49 |  |
| 10 | Fucose | 0.16 |  | -0.12 |  | 0.21 |  |
| 11 | Valerate | -0.08 |  | 0.02 |  | 0.52 | * |
| 12 | Lactate | -0.13 | * | -0.22 |  | 0 |  |
| 13 | Alanine | 0.03 |  | -0.23 |  | -0.16 |  |
| 14 | Thymine | 0.05 |  | -0.11 |  | 0.93 | ** |
| 15 | Acetate | 0.28 | * | -0.11 |  | 0.15 |  |
| 16 | N-acetyls of glycoproteins | 0.15 |  | -0.21 |  | -0.15 |  |
| 17 | Glutamate | 0.10 |  | -0.26 |  | 0.96 |  |
| 18 | Pyruvate | 0.14 |  | -0.4 | * | -1.01 | * |
| 19 | Succinate | -2.67 | ** | 3.34 | ** | 2.79 | ** |
| 20 | Glutamine | -1.83 | * | 1.37 | * | 1.35 | * |
| 21 | Methionine | 0.34 | * | -0.04 |  | -0.4 |  |
| 22 | Sarcosine | -0.04 |  | -0.01 |  | -0.18 |  |
| 23 | Aspartate | -0.25 |  | 0.11 |  | 0.37 | * |
| 24 | Trimethylamine | 0.00 |  | -0.29 |  | 0 |  |
| 25 | Lysine | 0.18 |  | -0.27 |  | -0.28 |  |
| 26 | Putrescine | 0.00 |  | -0.39 | * | -0.47 |  |
| 27 | Malonate | -0.09 |  | 0.11 |  | 0.26 |  |
| 28 | Choline | -0.21 |  | -0.55 | * | -0.32 |  |
| 29 | Taurine | 0.42 | * | -0.12 |  | -0.52 | * |
| 30 | Methanol | -0.01 |  | -0.73 | * | 0.15 |  |
| 31 | Glycine | 0.06 |  | 0.01 |  | -0.5 |  |
| 32 | Galactose | 1.09 | * | -1.09 | * | -1.96 | ** |
| 33 | Glucose | 0.54 | * | 0.14 |  | -0.39 |  |
| 34 | Ribose | 0.65 | * | -0.29 |  | -0.3 |  |
| 35 | Uracil | 1.03 | * | -1.22 | * | 0.17 |  |
| 36 | Urocanate | 0.05 |  | 0.2 |  | -0.1 |  |
| 37 | Fumarate | 0.12 |  | 0.03 |  | -0.27 |  |
| 38 | Tyrosine | 0.97 | * | -0.81 | * | -0.89 | * |
| 39 | Histidine | 0.07 |  | 0.27 |  | -0.72 | * |
| 40 | Phenylalanine | 0.86 | * | -0.84 | * | -0.97 | ** |
| 41 | Xanthine | 0.94 | ** | -1.06 | ** | -0.61 | * |
| 42 | Hypoxanthine | 1.33 | * | -0.9 | ** | 0.08 |  |
| 43 | Formate | 3.17 | *** | -0.03 |  | -2.16 | * |
| 44 | Nicotinate | 0.58 | * | -0.2 |  | -0.2 |  |

^a^ FC: Color coded according to the fold-change value; color coded according to log2 (FC), red represents increased and blue represents decreased concentrations of metabolites. Color bar.





^b^ p‐Values corrected by Benjamini–Hochberg methods were calculated based on a parametric Student’s t test or a nonparametric Mann–Whitney test (dependent on the conformity to normal distribution). * p < 0.05, ** p < 0.01 and *** p < 0.001.

Table.S4 Contrast of serum differential metabolites

| No. | Metabolites | MCAO VS Sham | | LM VS MCAO | | HM VS MCAO | |
| --- | --- | --- | --- | --- | --- | --- | --- |
|  |  | log_2_（FC） | *P* value | log_2_（FC） | *P* value | log_2_（FC） | *P* value |
| 1 | Lipid, fatty acid chains -CH_3_ | 0.06 |  | 0.07 |  | -0.01 |  |
| 2 | Leucine | 0.08 |  | -0.14 |  | -0.08 |  |
| 3 | Valine | 0.21 |  | -0.10 |  | 0.14 |  |
| 4 | Isoleucine | 0.27 |  | 0.15 |  | 0.55 | * |
| 5 | 3-Hydroxybutyrate | -0.78 |  | -0.01 |  | -0.89 |  |
| 6 | Lactate | -0.23 | * | 0.18 |  | -0.11 |  |
| 7 | Alanine | -0.05 |  | 0.04 |  | 0.12 |  |
| 8 | Lysine | -0.06 |  | -0.14 |  | 0.26 |  |
| 9 | Acetate | -0.30 |  | -0.08 |  | -0.21 |  |
| 10 | N-acetyls of glycoproteins | 0.06 |  | 0.09 |  | 0.11 |  |
| 11 | Lipid, fatty acid chains -CH_2_ | 0.25 |  | 0.24 | * | 0.02 |  |
| 12 | Glutamine | 0.35 |  | 0.25 |  | 0.01 |  |
| 13 | Malate | -0.01 |  | 0.21 |  | 0.55 | * |
| 14 | Creatine | -0.20 |  | -0.07 |  | 0.18 |  |
| 15 | Choline | -0.03 |  | -0.10 |  | -0.10 |  |
| 16 | Glucose | 0.12 |  | -0.03 |  | -0.19 |  |
| 17 | Methanol | 0.17 |  | -0.42 |  | 0.04 |  |
| 18 | Tyrosine | -0.37 | * | 0.41 |  | 0.31 |  |
| 19 | Phenylalanine | -0.52 |  | 0.08 |  | 0.33 |  |
| 20 | Tryptophan | -0.40 |  | 0.39 |  | 0.92 | * |
| 21 | Formate | -0.39 |  | 0.38 |  | 0.72 | * |

^a^ FC: Color coded according to the fold-change value; color coded according to log2 (FC), red represents increased and blue represents decreased concentrations of metabolites. Color bar.





^b^ p‐Values corrected by Benjamini–Hochberg methods were calculated based on a parametric Student’s t test or a nonparametric Mann–Whitney test (dependent on the conformity to normal distribution). * p < 0.05, ** p < 0.01 and *** p < 0.001.
